# Structure of the dimeric ATP synthase from bovine mitochondria

**DOI:** 10.1073/pnas.2013998117

**Published:** 2020-09-08

**Authors:** Tobias E. Spikes, Martin G. Montgomery, John E. Walker

**Affiliations:** ^a^The Medical Research Council Mitochondrial Biology Unit, Cambridge Biomedical Campus, University of Cambridge, Cambridge CB2 0XY, United Kingdom

**Keywords:** bovine mitochondria, dimeric ATP synthase, structure, Grotthus chain, torque generation

## Abstract

The structure of the dimeric ATP synthase from bovine mitochondria determined in three rotational states by electron cryo-microscopy provides evidence that the proton uptake from the mitochondrial matrix via the proton inlet half channel proceeds via a Grotthus mechanism, and a similar mechanism may operate in the exit half channel. The structure has given information about the architecture and mechanical constitution and properties of the peripheral stalk, part of the membrane extrinsic region of the stator, and how the action of the peripheral stalk damps the side-to-side rocking motions that occur in the enzyme complex during the catalytic cycle. It also describes wedge structures in the membrane domains of each monomer, where the skeleton of each wedge is provided by three α-helices in the membrane domains of the b-subunit to which the supernumerary subunits e, f, and g and the membrane domain of subunit A6L are bound. Protein voids in the wedge are filled by three specifically bound cardiolipin molecules and two other phospholipids. The external surfaces of the wedges link the monomeric complexes together into the dimeric structures and provide a pivot to allow the monomer–monomer interfaces to change during catalysis and to accommodate other changes not related directly to catalysis in the monomer–monomer interface that occur in mitochondrial cristae. The structure of the bovine dimer also demonstrates that the structures of dimeric ATP synthases in a tetrameric porcine enzyme have been seriously misinterpreted in the membrane domains.

## Significance

Adenosine triphosphate (ATP), the fuel of life, is produced in inner membranes of the mitochondria of eukaryotic cells by an embedded molecular machine with a rotary action, called ATP synthase. Single ATP synthases associate into dimers and form long rows, influencing the formation of characteristic cristae which change shape constantly. Our structure of bovine dimers has a wedge made of small proteins and specific lipids in the membrane domain of each monomer that imposes a range of acute angles on the central axes of the monomers, and a pivot between the wedges accommodates rocking motions of the machine accompanying catalysis and other movements that happen independently. It also throws light on how the membrane rotor is made to turn.

Mitochondrial ATP synthases occupy the inner membranes of the organelle and form dimers via specific interactions in their membrane domains. The dimers themselves are linked together in back-to-front rows along the edges of the cristae (1–3). The ATP synthases from bovine and *Saccharomyces cerevisiae* mitochondria are established paradigms for studying the structure, mechanism, and regulation of mitochondrial ATP synthases, and the bovine enzyme is a surrogate for the human enzyme. Each monomeric unit in the dimeric bovine enzyme described here is an assembly of 28 polypeptide chains of 17 different kinds, organized into a spherical catalytic globular domain that extends from the inner mitochondrial membrane (IMM) into the mitochondrial matrix, attached to an intrinsic membrane domain by a central stalk and a peripheral stalk (PS) (4). This membrane-bound enzyme is a machine that transmits by a rotary action the potential energy of the transmembrane proton motive force, generated by respiration, into the catalytic sites in the extrinsic domain. The membrane-bound rotor consists of a ring of eight identical c-subunits in close association with a single static a (or ATP-6) subunit, attached to the asymmetrical central stalk (subunits γ, δ, and ε) (5, 6) which extends from the membrane domain and penetrates into the extrinsic globular catalytic domain along its central axis. As it rotates, the asymmetrical central stalk brings about structural changes in the three catalytic sites, found mainly in each of the three β-subunits, which alternate with three noncatalytic α-subunits in the spherical extrinsic domain (4, 7). These structural changes lead sequentially at each catalytic site to the binding of the substrates ADP and phosphate, followed by the formation and then the release of ATP. The PS, made from the subunits oligomycin sensitivity conferral protein (OSCP), b, d, F_6_, and the membrane extrinsic region of ATP8, link the external surface of the catalytic domain to the a-subunit in the membrane domain (8, 9). Together, the α_3_β_3_-domain, the PS, and subunit a constitute the enzyme’s stator against which the rotor turns. Subunits e, f, g, A6L (or ATP8), and j (or the 6.8 proteolipid) also contribute to the membrane domain of the PS (10–12), and in the dimeric complex some of them are involved in forming the interface between monomeric complexes (12). Another subunit known previously as diabetes-associated protein in insulin sensitive tissue (11), but here renamed “k” to be consistent with the yeast ortholog (12, 13), may be involved in forming links between dimer units in the rows of dimers (13, 14). Over a period of about 15 y from 1994, a mosaic structure representing about 85% of the monomeric complex was built up by solving structures of separate domains by X-ray crystallography (5, 9, 15–25). The missing 15% represented part of the membrane domain. An intact bacterial ATP synthase was finally solved by X-ray crystallography in 2015 (26). With the advent of electron cryo-microscopy (cryo-EM), from 2015 onward a range of structures of intact monomeric ATP synthases from bacteria (27–29) and chloroplasts (30), and of dimeric ATPases from various mitochondria have been described at a range of resolutions (6, 31–36), as well as the dimeric membrane domain from the enzyme from *S. cerevisiae* (12). These structures have confirmed the widespread conservation of the central structural and mechanistic features of the enzyme established mainly in the bovine enzyme, and the knowledge that the stoichiometry of the c-ring varied according to species from 8 to 17 (5, 37–39). They also demonstrated extreme variation in the subunit composition, sequences, structure, and apparent rigidity of the PS in the various enzymes. The atomic models of the dimeric bovine ATP synthase described here provide evidence of the operation of a Grotthus mechanism of proton translocation (40) in the inlet half channel of the transmembrane proton pathway through the membrane domain. The structures provide insights into the architecture and mechanical properties of the PS (35) and describe wedge structures in the membrane domains of each monomer that allow the monomer–monomer interfaces to pivot during catalysis and to accommodate other changes not related directly to catalysis that occur in mitochondrial cristae. The bovine structure shows that the structures of the membrane domains of porcine ATP synthases in a tetrameric complex have been seriously misinterpreted (41).

## Results and Discussion

### The Structure of Dimeric Bovine ATP Synthase.

The dimeric enzyme, inhibited by residues 1 to 60 of regulatory protein IF_1_, was purified in the presence glycodiosgenin, Brij-35, and phospholipids (*SI Appendix*, Fig. S1), and its structure was determined by cryo-EM of single particles (*SI Appendix*, Fig. S2). Three datasets of 4,267; 2,238; and 4,096 dose-fractionated exposures were curated manually and auto-picked to provide 233,330 initial particle coordinates in 9,110 curated micrographs, 176,710 of them remaining after iterative rounds of two-dimensional classification and selection of particle subsets. Particles in each dataset were merged, and structures of the monomeric enzyme were derived according to *SI Appendix*, Scheme S1. The resolution was increased by exploiting the pseudoc2 symmetry of the dimer, yielding 253,473 monomer particles with 101,165, 90,850, and 61,458 in rotational states 1, 2, and 3, respectively, with corresponding resolutions of 3.2, 3.3, and 3.5 Å. Focused local refinement (*SI Appendix*, Scheme S2) of the rotor (c_8_-ring plus subunits γ, δ, and ε), the stator (subunits OSCP, b, d, and F_6_ and associated supernumerary subunits e, f, and g) and the membrane domain (subunits a, A6L, b, d, e, f, g, j, k, and the c_8_-ring) improved the quality of the maps and the resolutions in these regions to 3.5 to 3.7, 4.2 to 6.0, and 3.6 Å.

**Fig. 1.**
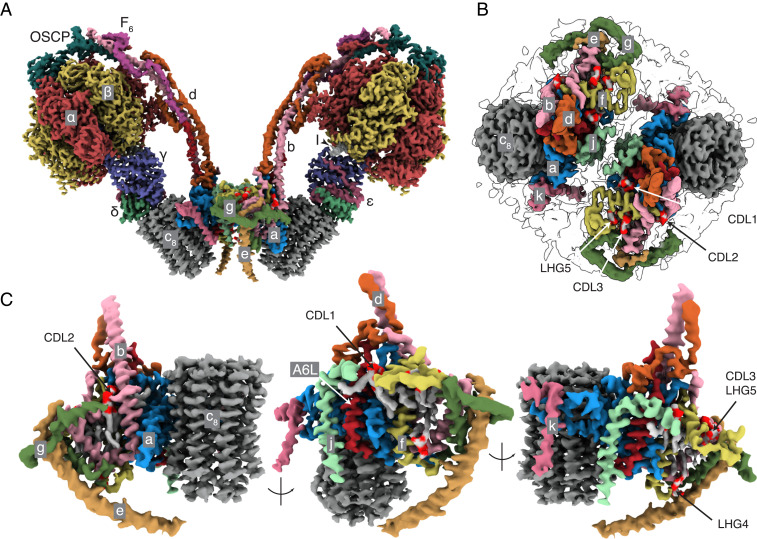
Structure of dimeric bovine ATP synthase. The state 1–state 1 dimer is depicted with each monomer inhibited by residues 1 to 60 of the inhibitor protein IF_1_, states 1, 2, and 3 being the three rotational states of the F_1-_domain in each monomer relative to the PS. (*A* and *B*) The densities derived by cryo-EM represented as volume zones within 2.0 and 1.8 Å, respectively, of the fitted atomic model, viewed in *A* from the side (orthogonal to the plane of the IMM) and in *B* from inside the mitochondrial matrix toward the interface between monomers. In *A*, the detergent micelle, and in *B*, the F_1_-domain and the membrane extrinsic region of the PS, have been removed for clarity. The inhibitor protein is labeled I. In *B*, the outline of the detergent micelle is indicated by gray lines. (*C*) Arrangement of subunits in the membrane domain of the monomer. The α-, β-, γ-, δ-, and ε-subunits of the F_1_-catalytic domain are red, yellow, blue, indigo, and green, respectively, with the central stalk (subunits γ, δ, and ε) attached to the c_8_-ring (dark gray) in the membrane domain in contact with subunit a or ATP6 (cornflower blue). The PS subunits OSCP, b, d, and F_6_ are teal, light pink, orange, and magenta, respectively, and the A6L subunit is brick red. In the region of the monomer–monomer interface, subunits e, f, g, j, and k are khaki, straw yellow, forest green, sea-foam green and dark pink, respectively. Cardiolipin (CDL) and phosphatidyl-glycerol (LHG) phosphate headgroups are scarlet, and the acyl chains are gray. See also Movie S1.

**Fig. 2.**
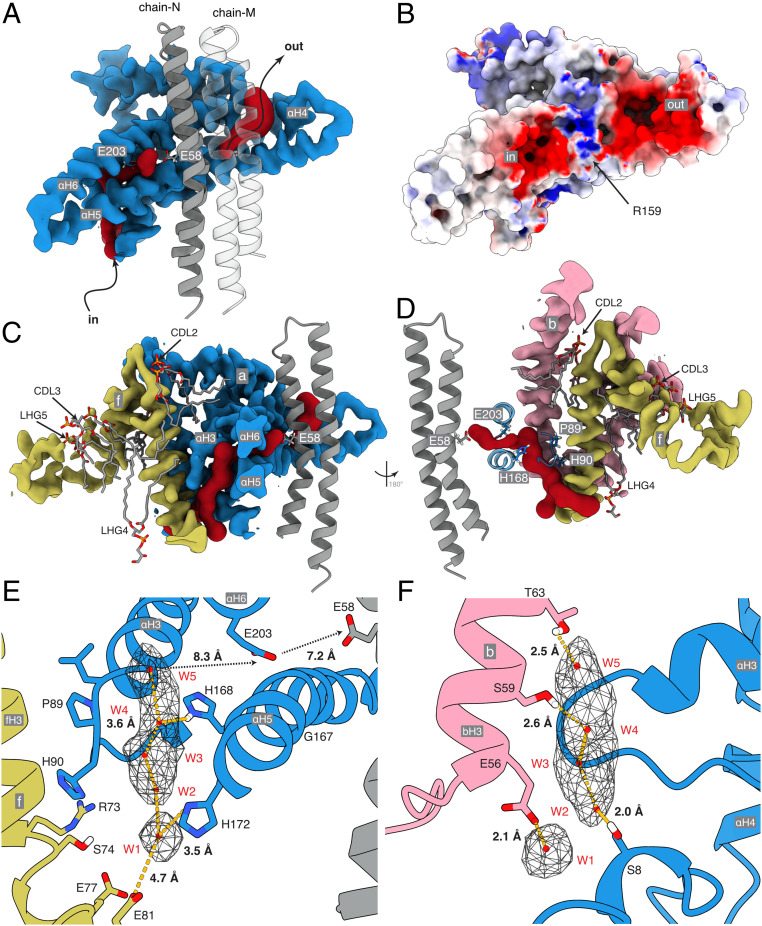
Proton half-channels in bovine ATP synthase with a Grotthus water chain in the inlet. (*A* and *B*) Cross-section of part of the a-subunit (blue) plus a two adjacent c-subunits (gray) viewed from inside the c_8_-ring. (*A*) The inverted L-shape of the inlet channel and the tilted outlet half-channel (both red) determined with CAVER. (*B*) The electrostatic potential surface of the inlet and outlet half-channels separated by a region of positive charge around aArg-159. The electrostatic potential of the molecular surface, calculated with the DELPHIpKa web-server (62, 63) at a salt concentration of 150 mM, a pH of 7.4, and otherwise default parameters, is shown with positively and negatively and positively charged surfaces in blue and red, respectively, and the c_8_-ring in transparency. The range of charge potentials is −5 (red) to +7 (blue). (*C* and *D*) The topology of the inlet cavity with a vertical cross-section of the membrane domain of the proteins and adjacent phospholipids CDL2, CDL3, LHG4, and LHG5. (*E* and *F*) Detailed views of the inlet channel with charged and polar residues available for hydrogen bonding a Grotthuss chain of five water molecules W1 to W5 (red spheres) suggested by the unmodeled density (in mesh). The view in *E* is equivalent to that in *C*. *F* is viewed through the channel from cGlu-58 toward the loop of subunit a containing aPro-90 (*D*). Distances between H-bond donors and acceptors and water molecules are shown. Between W1 and W5 the distances are 4.1, 3.2, 2.6, and 4.0 Å, respectively. The Grotthuss chain leads to aGlu-203 and then to cGlu-58 possibly via other as-yet-undefined water molecules.

**Fig. 3.**
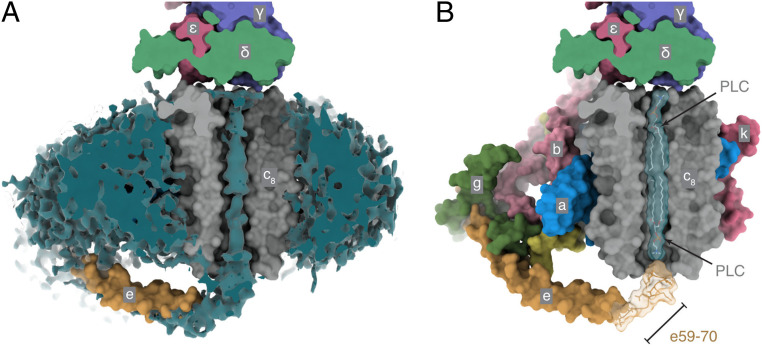
Occupancy of the inner cavity of the c_8_-ring. (*A*) Cross-section of the solvent-excluded surface of the model of the c_8_-ring and e-subunit of the membrane domain, the foot of the central stalk, and the unmodeled density (teal), which is mainly the detergent-lipid micelle surrounding the protein, plus density extending beyond residue e58 toward the opening of the central cavity of the c_8_-ring. (*B*) Cross-sectional view of the solvent-excluded surface of the membrane domain. The unmodeled region (khaki transparency) probably represents residues e59 to the C-terminal residue e70. It is conceivable that a brief span of the extreme C-terminal region penetrates a short distance into the cavity of the c_8_-ring and that this region is occupied by the C-terminal tail of subunit e plus a phospholipid (PLC). The density inside the c_8_-ring is weak and rotationally averaged and probably represents PLC molecules (*SI Appendix*, Fig. S13). A second PLC is shown occupying the upper region of the cavity.

In the structure of the dimeric bovine complex (Fig. 1 and Movie S1), all subunits and sequences were assigned unambiguously (*SI Appendix*, Fig. S3) with improvement in the models for the γ-subunit in the F_1_-domain and in the OSCP in the PS (*SI Appendix*, Table S1). The only significant remaining structural ambiguity is in the region of the PS around where the C-terminal regions of F_6_ and A6L meet (*SI Appendix*, Fig. S4). In the membrane domain, the detailed structures of the a-subunit and c_8_-ring and the proton pathway that they provide were improved (Fig. 2), as was the complex “wedge” involving the b-subunit in the peripheral stalk and associated supernumerary subunits e, f, g, j, k, and A6L. Three cardiolipins (CDL1–3) and two other phospholipids designated phosphatidyl-glycerols (LHG4–5) are specifically bound in each wedge (*SI Appendix*, Fig. S5). Because of the quality of the density, there remains uncertainty about the identity of LHG4–5, and either or both could conceivably be cardiolipin molecules. In the dimeric ATP synthase from the single-cell flagelate *Euglena gracilis*, several cardiolipin molecules were identified at the interface between monomers, two of them in similar locations to CDL1 and CDL2, although the architecture, subunit compositions, and mechanisms of dimerization differ considerably from the bovine dimer (35). The inhibition of ATP hydrolysis in the isolated catalytic F_1_-domain arrests the rotary cycle at the catalytic dwell, and in structures of the inhibited bovine F_1_-IF_1_ complex, the rotary cycle has been arrested at 27° to 32° after the pause in the rotary cycle preceding the release of phosphate (25). In the current structure, the postphosphate release angles are 28.8°, 26.2°, and 25.5° in states 1 to 3, respectively. Therefore, the rotational angle in the F_1_-domain is not influenced significantly by interactions with the additional components of the intact enzyme that are absent from the isolated F_1_-domain. The structure of the dimeric bovine ATP synthase is consistent with chemical cross-linking data (42) (*SI Appendix*, Fig. S6), and the attribution of subunits is the same as in the dimeric yeast membrane domain (12). The bovine structure differs significantly from those of dimeric complexes in a tetrameric form of the closely related porcine enzyme (41) (*SI Appendix*, Figs. S7 and S8) where subunit j was misidentified as subunit k (*SI Appendix*, Figs. S9 and S10), and subunit k as a hypothetical protein (*SI Appendix*, Figs. S11 and S12). Also, porcine subunit j was built as a polyalanine α-helix in the central cavity of the c_8_-ring, whereas the cavity is most likely to be occupied by lipids (Fig. 3 and *SI Appendix*, Fig. S13; see below), and other differences occur in the structures of subunits b, c, e, f, g, and A6L (*SI Appendix*, Figs. S14–S18).

### The Peripheral Stalk.

The PS is an essential component of the enzyme’s stator linking the α_3_β_3_-sphere of the F_1_-catalytic domain to the membrane-associated wedge and the adjacent a-subunit into the stator subcomplex against which the rotor turns. During catalysis, it prevents the dissociation of the α_3_β_3_-domain from the central stalk by clamping it in position from above, and it resists the rotational torque of the central stalk, preventing the α_3_β_3_-domain and the rest of the stator domain from following the direction of rotation. It is evident from Movie S2 that the PS has four mechanical components. A cap provided by the N-terminal domain of the OSCP is linked to the N-terminal extensions of the three α-subunits and adjusts its connections in concert with the rocking of the α_3_β_3_-domain during a catalytic cycle (*SI Appendix*, Fig. S19). The cap is connected by a universal joint (OSCP residues 112 to 116) to the C-terminal domain of the OSCP (residues 175 to 190) (*SI Appendix*, Figs. S20–22). Its role is to accommodate the PS to the up–down and sideways rocking motions of the α_3_β_3_-domain during catalysis caused by the asymmetric rotor (Movie S3). This universal joint is linked to a rigid rod *∼*150 Å in length consisting largely of parallel (not coiled) α-helical sections of subunits b (residues 91 to 186 and 190 to 214), d (residues 24 to 43, 53 to 59, 63 to 75, 84 to 122, and 132 to 138), and F_6_ (residues 9 to 24 and 33 to 50), augmented in the lower region by the C-terminal region of A6L (residues 30 to 66) (Fig. 1*A* and *SI Appendix*, S4 and S23 *A*–*C*). The rigid rod is connected to the membrane domain of the stator by a hinge at residues 73 to 90 of bH3 (the penultimate C-terminal α-helix of subunit b), which remains relatively immobile as bH3 and bH4, and associated subunits d, F_6_, and OSCP are displaced laterally by 9.9 and 13.1 Å in states 1 and 3, respectively, measured relative to state 2 (*SI Appendix*, Fig. S23). In contrast to the *Escherichia coli* ATP synthase, where the PS is simpler and consists of a right-handed α-helical coiled-coil provided by the two identical b-subunits, additional displacements perpendicular to the lateral motion of the PS (toward the central axis of the rotor) have been observed (27, 29), but there are no such movements in the catalytic cycle of the bovine dimer. Rather, in the state 3–state 1 transition, the OSCP expands as the PS moves outward from the F_1_ domain (Movie S3). In bovine dimer structures representing the gamut of catalytic states, the two membrane domains do not superimpose, but they pivot, as we describe elsewhere. Thus, these lateral displacements of the peripheral stalk brought about by the rotation of the asymmetrical central stalk are transmitted to the membrane domain, with a reduction in the displacement of the catalytic domain relative to the bacterial enzyme. In the dimeric and oligomeric assemblies found along the apices of the mitochondrial cristae, this reduced displacement may provide a damping effect and increase the efficiency of the enzyme. Some degree of motion of the catalytic domain must be permitted to allow efficient conversion of the rotational torque of the central stalk into conformational changes in the catalytic subunits, but without wasting energy by unneeded displacement of the extrinsic portion of the enzyme. The flexibility of the peripheral stalk effectively meters this balance, preventing free rotation of the catalytic domain, accommodating the rocking motions induced by the asymmetric central stalk, and damping displacement of the membrane domain helping to keep the a-subunit in place.

### The Proton Pathway.

The roles of the a-subunit and c-ring in providing the proton pathway through the IMM have been described before (6, 12, 28, 30, 31, 33, 35, 43, 44). During ATP synthesis, an inlet half-channel passing through α-helices aH5 and aH6 in the a-subunit tilted at 30° to the plane of the membrane provides ingress for protons to negatively charged Glu-58 residues in c-subunits (or cGlu-58 residues) located at the approximate midpoint of the membrane. The protons are carried through the lipid bilayer on the neutralized γ-carboxyl of cGlu-58 by the rotation of the c_8_-ring to the outlet half-channel where they are released by the positively charged aArg-159 into the outlet half-channel, provided by the tilted α-helices in the a-subunit. In the current structure (Fig. 2), the inlet and outlet channels are defined clearly, and the c_8_-bearing surface of the a-subunit has negatively charged regions corresponding to the two half-channels (Fig. 2*B*). The half-channels are separated by a positively charged region provided by the highly conserved aArg-159 (and others) that prevents, probably by charge repulsion, leakage of protons from inlet to outlet and promotes deprotonation of cGlu-58 in the outlet channel to drive the rotation of the c_8_-ring in the synthesis direction. Details have emerged relating to the inlet half-channel especially, which has a negatively charged entrance to attract hydronium ions (*SI Appendix*, Fig. S24). The half-channel is formed from elements of the a-, b-, and f-subunits with the loop between aH3 and aH4 (containing residues aPro-89 and aHis-90) and together with the C-terminal region and transmembrane α-helix of fH3 (residues 56 to 74) providing its “back." The “front” is formed from regions of aH5 and aH6 (which contains the conserved aGlu-203 residue), and the second transmembrane α-helix of the b-subunit, bH3, closes this side of the channel, sealing it from the aqueous environment and the surrounding membrane (Fig. 2 *C* and *D*). Lipids also contribute significantly (Fig. 2 *C* and *D* and *SI Appendix*, S5 and S25). Cardiolipins CDL2 and CDL3 and an acyl chain of LHG4 occupy cavities blocking solvent access of water at interfaces with the transmembrane α-helices of subunits b and f, thereby preventing proton leakage via the back of the a-subunit. CDL2, and the acyl chains of CDL3 and LHG4 at the interface between bH3 and fH3, reinforce specific spatial relationships between α-helices in the half-channel that otherwise might “collapse” into the unoccupied spaces. The route for protons from the intermembrane space of the mitochondria leads to the highly conserved aGlu-203, located where the solvent channel traverses through the tilted α-helices aH5-aH6 and thence toward the c_8_-rotor (Fig. 2*A*). Several polar and charged residues in the inlet half-channel are in position to hydrogen-bond a network of five water molecules, W1 to W5 (Fig. 2 *E* and *F*), suggested by unmodeled density, and to transfer protons by a Grotthus mechanism. Immediately below the conserved aGlu-203 in aH5, a small aperture between the solvent cavities on either side of aH4 and aH5 allows protons to be transferred between two adjacent water molecules (Fig. 2 *C* and *E*) in a final step. The distances between bGlu-56, bSer-59, and bThr-63 to water molecules W1, W3, and W4, respectively, are suggestive of strong hydrogen bonds (Fig. 2*F*), while those between aSer-8, aHis-168, and aHis-172 and W2, W3, and W1, respectively, suggest weaker interactions (Fig. 2 *E* and *F*). The relatively large distances between W5 and aGlu-203, and between aGlu-203 and cGlu-58, in the final steps of the proton transfer, may suggest the involvement of other as-yet-undefined water molecules. Alternatively, the transfer after W5 could be mediated by diffusion of free hydronium ions. The proximity of aHis-168 to this apparently empty region of the half-channel suggests its participation. In a structure of dimeric ATP synthase from *Polytomella* (35), an unspecified metal ion coordinated by two histidine residues was modeled near to the position of W1 in the bovine structure, but only one of these histidines, aHis-172, was conserved in the bovine enzyme. In addition to proffering a clear pathway for the transfer of protons to the c_8_-ring, the Grotthus chain presents an apparent mechanism to transmit the full energetic potential of the proton motive force (pmf) into torque to drive the rotor by providing a stretch of high permittivity medium to transpose the vertical membrane potential into a horizontal potential. The strong positive electric field at the entrance to the inlet channel is conducted toward the negatively charged carboxyl of the cGlu-58 at the midpoint of the membrane, and, as would be required for ATP synthesis, driving the c_8_-ring forward in the appropriate direction with the full force of, but perpendicular to, the electro-chemical proton gradient. While a notional Brownian ratchet mechanism ensures the directionality of rotation of the c_8_-ring (45), coupling the ring directly to the membrane potential via the Grotthus water chain is a mechanism for delivery of the full proton motive force to the ring generating the torque required to turn the ring and the entire rotor against the resisting forces of the catalytic domain.

In the more open funnel-shaped outlet half-channel, strong density near aArg-159, with the hydroxyl moiety of aTyr-221 pointing toward it, could be a specifically bound water molecule, with distances of *∼*2 Å to each of the hydrogen bond acceptors of aArg-159. The bound water molecule could accept the proton leaving cGlu-58, probably via freely diffusing water as the distance between cGlu-58 and aArg-159 is too large for direct transfer. Release of protons into the matrix may occur via a chain of waters, as in the inlet channel; by successive transfers via residues in the upper region of the outlet (with aGlu-145, aHis-127, aHis-223, and aAsp-224 providing hydrogen bonds); or simply by diffusion into the bulk solvent. If so, the upper part of the outlet channel would provide a sufficiently negative environment to maintain a pool of nearby hydronium ions, rather than providing a specific exit pathway. In synthesis, and therefore in the presence of a pmf, the charge separation across the inner mitochondrial membrane should act to drive protons away from the outlet channel. During hydrolysis, where torque and rotation are generated in the catalytic domain driven by the energy released by ATP hydrolysis, the funnel shape should help to collect protons into the channel and lead them to cGlu-58 so they can be pumped back into the mitochondrial matrix via the inlet channel. The arrangement of a specific channel delivering energy to the rotor and a rather nonspecific proton exit pathway is reminiscent of the arrangement of proton pathways in cytochrome *c* oxidase where a specific channel delivers protons via a Grotthus mechanism to the binuclear center to provide the energy to drive the reduction of dioxygen, whereas the exit pathway for protons into the mitochondrial matrix is nonspecific (46).

### Contents of the c_8_-Rotor Ring.

The central cavity of the c_8_-ring contains a poorly defined, noisy density observed also in the porcine (41) and yeast c-rings (12) (Fig. 3*A* and *SI Appendix*, S13 *A*–*E*). Here, the upper half of this density has been attributed to a mobile lipid (including possibly coenzyme Q_10_) (47), and the lower half has been ascribed to another mobile lipid and the C-terminal region of the e-subunit, which extends toward the central cavity on the inter-membrane space (IMS) side of the membrane and could just penetrate into the cavity (Fig. 3*B*). Residues eArg-58 and eLys-70 could interact with a ring of aspartate residues at the bottom of the c_8_-ring. Alternatively, eGlu-62, eGln-64, eGlu-65, and eAsp-66 could bind and neutralize the charge of a positive lipid headgroup in the otherwise highly nonpolar internal environment of the IMS half of the c-ring cavity to tether the C terminus of the e-subunit while allowing unimpeded rotation of the c_8_-ring. In contrast, in the dimeric yeast ATP synthase, the C-terminal region of the e-subunit points outward into the IMS in the direction of the c_10_-ring of the second monomer and does not enter the cavity (12). The matrix side of the bovine cavity has a net positive electrostatic potential provided by the ring of eight trimethyllysine residues at position 43. In all eight bovine c-subunits, the density supports this modification (*SI Appendix*, Fig. S26). The surface of the internal cavity of the c_8_-ring is almost entirely hydrophobic except for the side chains of residues cThr-27 and cSer-31, and the cavity is hourglass-shaped, narrowing from a 14.2-Å diameter at the entrances to 10.4 Å at the approximate midpoint of the membrane. These characteristics are incompatible with the suggestion that the cavity provides a nonspecific aqueous channel for calcium ions known as the mitochondrial permeability transition pore (48–50) that can also transmit polyethylene glycols with sizes up to PEG 1000 (51).

### The Monomer–Monomer Interface.

**Fig. 4.**
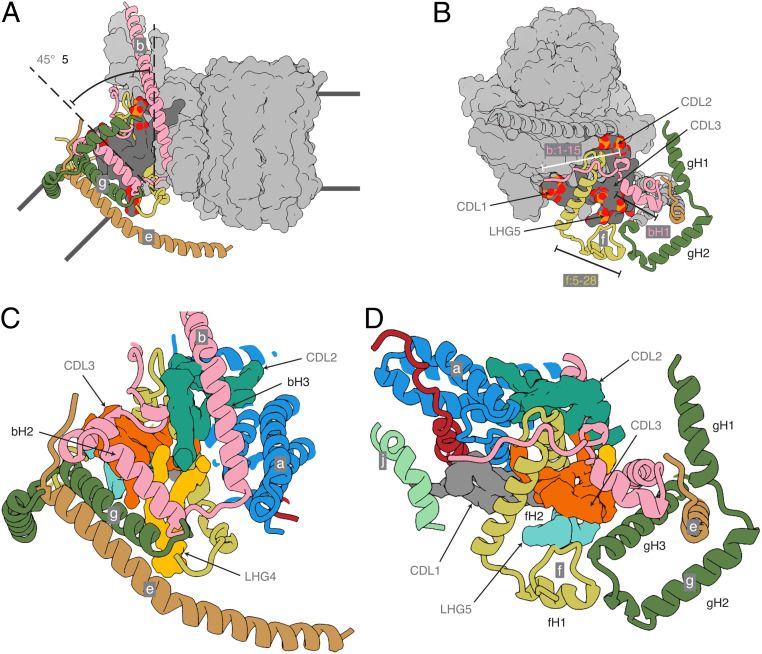
The structure of the wedge at the monomer–monomer interface in dimeric bovine ATP synthase. Subunits b, e, f, and g are pink, khaki, straw-yellow, and forest green, respectively, and subunits a, j, k, A6L, and the c_8_-ring are depicted as a light-gray solvent-excluded surface. The dark-gray solvent-excluded surface represents bound lipids (CDL1, CDL2, CDL3, and LHG5) with the phosphate and oxygen atoms of the head groups in orange and red, respectively. The black lines indicate the approximate boundaries of the lipid bilayer. (*A*) Side view of the membrane domain of the monomer showing the tilted arrangement of the transmembrane α-helices of subunits b, e, and g. (*B*) Top view demonstrating how the unique N-terminal topologies of subunits b, e, and g, lying in the plane of the matrix leaflet of the membrane, form part of a wedge that similarly reinforces the curvature. In addition, the transmembrane α-helix of subunit g is tilted toward the center of the dimer complex by *∼*40°. (*C* and *D*) Magnified views of the same orientations of the wedge with the cryo-EM densities of the identified lipids CDL1, CDL2, CDL3, LHG4, and LHG5 in gray, turquoise, orange, yellow, and cyan, respectively. Subunits, a, A6L, and j are cornflower blue, brick red, and sea-foam green, respectively.

The structure explains the roles of supernumerary subunits e, f, and g and of α-helices bH1 and bH2, which are not present in the monomeric ATP synthases from bacteria and chloroplasts. Together with the transmembrane domain of bH3, these elements form a wedge (Fig. 4), with the upper surface on the matrix side of the membrane subtending an angle of *∼*45° in the membrane domain of each monomer. This angle is imposed by the α-helices bH2 and bH3, which provide the wedge with a protein skeleton, with bH1 sitting on top on the matrix side. The position of bH2 is augmented by the transmembrane segment of subunit e (residues 10 to 25) and by a second transmembrane α-helix, gH3 (residues 69 to 93). The top of the wedge on the matrix side consists of four amphipathic α-helices: gH1 (residues 20 to 36), gH2 (residues 42 to 60), fH1 (residues 16 to 25), and fH2 (residues 30 to 48). This topology is supported by residues 1 to 15 of subunit b, which bind to residues 128 to 131 of subunit d in the peripheral stalk. Amphipathic α-helices fH1 and fH2, bH1, and gH2 are gathered around the headgroups of CDL3 and LHG5, and CDL3 and LHG5 occupy a protein void in the matrix leaflet of the membrane. CDL1, CDL2, and LHG4 fill internal protein voids, sealing the membrane and adding stability to the wedge (Fig. 4). The negative charges of the cardiolipins are compensated by the positive charges of lysine and arginine residues adjacent to the lipid head groups (*SI Appendix*, Fig. S5). For example, CDL3 is coordinated by residues fArg-43 and fLys-47, and CDL1 by residues A6L-Lys-27, A6L-Lys-30, and jLys-8. The acyl chains of the lipids all occupy the hydrophobic interior of the membrane domain. These resolved lipids are probably incorporated at specific, and as-yet-undefined, points in the process of assembly of the enzyme, and there are likely to be others bound at the interface that were not resolved due to the placement of the monomeric membrane domain masks used for focused local refinement (*SI Appendix*, Fig. S25).

**Fig. 5.**
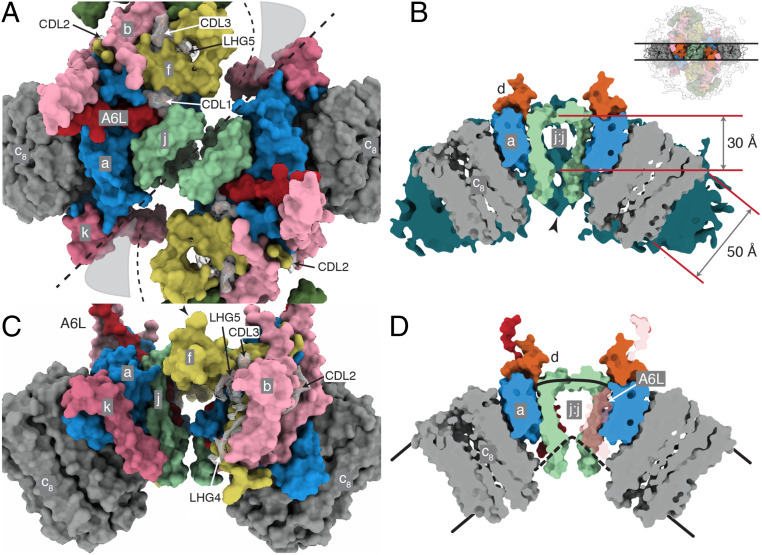
The monomer–monomer interface in the membrane domain of dimeric bovine ATP synthase. A composite model of the dimeric membrane domain at 3.6-Å resolution is shown. (*A* and *B*) The membrane domain viewed from between the two peripheral stalks and the orthogonal view, respectively. Subunits e and g, not involved in monomer–monomer contacts, have been removed to expose the protein–protein interface. The thick dashed line denotes the interface between the monomers, and the thinner dashed lines indicate regions between them lacking protein–protein interactions. (*C*) Cross-sectional view of the membrane domain of the dimeric bovine ATP synthase. The positions of the near and far clipping planes used to construct the cross-section are indicated in the *Inset* at the top. The molecular surfaces of the subunits are shown plus the detergent-lipid micelle (teal). For clarity, only subunits a, j, d, and the c_8_-ring are shown. The approximate thicknesses of the membrane in different regions of the membrane domain are indicated in ångströms. In the region of the interface between monomers, which is situated at the apex of the cristae, the membrane thins by ∼20 Å. The C-terminal regions of both j-subunits protrude into the IMS. Additional density connecting the protomers was observed in several of the dimer reconstructions at lower resolutions, as indicated by the black arrowhead. The boundary of the lower leaflet of the membrane lies above this interaction. (*D*) The same view of the membrane domain of the bovine complex with the micelle removed. In *D*, the A6L subunit is in brick red transparency. Its N-terminal residues define the approximate lower boundary of the membrane toward the c_8_-ring. This approximate boundary is indicated by the dashed black line.

A striking feature of the monomer–monomer interface region, which was inferred approximately from a catalytically averaged consensus reconstruction (*SI Appendix*, Scheme S1), is that the thickness of the membrane is reduced to *∼*30 Å from the usual value of *∼*50 Å. The intersection of the lower boundary of the membrane in the small cavity between the transmembrane α-helices of subunit j forms a sharp angle and the lower leaflet joins with the N-terminal hook (residues 1 to 7) of subunit A6L, the N terminus of subunit a (residues 1 to 15), and the C-terminal extension of subunit f (residues 74 to 87), forming the lower surface of the enzyme (Fig. 5 *C* and *D*). This lower surface contains the entrance to the proton inlet channel, with the charged residues aGly-3, aAsn-4, aThr-7, and aSer-8 in the N-terminal region and in the C-terminal region fGly-77, fHis-80, and fGly-81 providing a negative electrostatic potential amid a predominantly positively charged surface that should act to guide protons (as H_3_O^+^) to the inlet and subsequently into the channel toward aHis-172 (*SI Appendix*, Fig. S24). This thinning of the IMM facilitates its curvature where the two monomers are associated at the apices of the mitochondrial cristae. Although it has been demonstrated that ATP synthase dimers, and self-assembled oligomers formed from them, can induce bending in proteoliposomes in vitro (3, 52, 53), it is not known whether this effect is responsible for, or directly involved in, the production of cristae. If not, an alternative explanation is that the preformed dimers localize to the region of high positive curvature at the cristae tips. It has been proposed that a similar mechanism would also promote nonspecific oligomerization independently of defined protein interactions at the interface (although these interactions do exist) (3).

## Materials and Methods

Dimeric ATP synthase inhibited with residues 1 to 60 of the inhibitor protein IF_1_ was extracted from bovine mitochondria and purified in the presence of glycodiosgenin, Brij-35, and phospholipids. The purified enzyme was applied to electron microscopy grids, and high-resolution electron cryo microscopy data were collected with Titan Krios instruments. Three datasets were merged, and the structure of the monomeric ATP synthase was determined. The resolution of the reconstructions was increased by exploiting the pseudoc2 symmetry of the dimer. The quality of the map and the resolution of specific regions were improved by focused refinement of the stator, PS, and membrane domain. Fourier shell correlation curves and local resolution estimations were calculated with RELION. Model building into focused maps was performed with COOT (54) and real space refinement with PHENIX (55–57). Lipids were modeled into density with ISOLDE (58). The starting model comprised the crystal structures of subdomains of bovine ATP synthase. Model geometry and density fit validation was performed by MolProbity (59, 60) and EMRinger (61), respectively. For further details, see *SI Appendix*.

## Supplementary Material

Supplementary File

Supplementary File

Supplementary File

Supplementary File

## Data Availability

Protein models and electron density map data have been deposited in the Protein Data Bank and the Electron Microscopy Data Bank under the following accession numbers: 6YYO (EMD-11001), 6Z1R (EMD-11039), 6Z1U (EMD-11040), 6ZBB (EMD-11149), 6ZG7 (EMD-11195), 6ZG8 (EMD-11196), 6ZIK (EMD-11227), 6ZIQ (EMD-11228), 6ZIT (EMD-11229), 6ZIU (EMD-11230), 6ZPO (EMD-11342), 6ZQM (EMD-11368), 6ZQN (EMD-11369), 6ZMR, 6ZNA.
