# Supplementary material for: Structure of the dimeric ATP synthase from bovine mitochondria
Source: Proc Natl Acad Sci U S A. 2020 Sep 8;117(38):23519–26. doi: 10.1073/pnas.2013998117 (PMC7519299; doi:10.1073/pnas.2013998117)
Supplement: Supplementary File [file pnas.2013998117.sapp.pdf]

PNAS
www.pnas.org

Supplementary Information for

Structure of the dimeric ATP synthase from bovine mitochondria

Tobias E. Spikes, Martin G. Montgomery and John E. Walker
The Medical Research Council Mitochondrial Biology Unit, University of Cambridge, Cambridge Biomedical Campus, Hills Road, Cambridge CB2 0XY, United Kingdom

Corresponding author: John E. Walker
Email: walker@mrc-mbu.cam.ac.uk

This PDF file includes:

Supplementary text
Figures S1 to S26
Tables S1 to S3
Legends for Movies S1 to S3
SI References

Other supplementary materials for this manuscript include the following:

Movies S1 to S3

www.pnas.org/cgi/doi/10.1073/pnas.2013998117

Supplementary Materials and Methods.

Purification of dimeric ATP synthase. Bovine heart mitochondria (1) were freed of bound inhibitor protein, IF_1, by washing the membranes with an alkaline phosphate buffer (50 mM disodium hydrogen phosphate, 100 mM sucrose and 0.5 mM EDTA). The washed membranes (765 mg of protein) were extracted with a solution of digitonin and dodecylmaltoside (DDM) at detergent:protein ratios (w/w) of 1.2 g/g (0.92%, w/v) and 1.0 g/g (0.76%, w/v), final concentrations, respectively. The suspension (50 ml) was stirred for 30 min at 20ºC, and then centrifuged (20 min; 24,000 x g; 20ºC). To the supernatant were added I1-60His (recombinant residues 1-60 of bovine IF_1 with a His_6 C-terminal tag (1-2 mg), plus buffer (1500 µl) consisting of 400 mM Tris, pH 7.3, 200 mM ATP and 200 mM magnesium sulfate. The solution was incubated at 37°C for 15 min with 1500 µl of buffer being added every 5 min. Then the sample was centrifuged (10 min; 24,000 x g; 20ºC), and NaCl and neutralized imidazole were added to the supernatant (final concentrations 150 mM and 25 mM, respectively). The resulting solution was filtered through a cellulose acetate membrane (0.22 µm pores), and the filtrate was applied to a column of HisTrapTM HP nickel-Sepharose (5 ml; GE Healthcare) equilibrated in a buffer containing 20 mM Tris, pH 7.4, 150 mM NaCl, 2 mM ATP, 2 mM MgSO4, 10% glycerol, 0.1% (w/v) glyco-diosgenin (GDN) and 0.1 mg/ml of an aqueous suspension of 1-palmitoyl-2-oleoyl-sn-glycero-3-phosphocholine (POPC), 1-palmitoyl-2-oleoyl-sn-glycero-3-phosphoethanolamine (POPE), 1-palmitoyl-2-oleoyl-sn-glycero-3-[phospho-rac-(1-glycerol) (POPG), and bovine heart cardiolipin (CL) (3:1:1:1, by wt). The column was eluted at a flow rate of 1 ml/min with buffer (50 ml) containing a linear gradient of imidazole from 25-500 mM. The UV absorption of the effluent was monitored at 280 nm and fractions were analysed by SDS-PAGE (2) and BN-PAGE with 4-16% Bis-Tris polyacrylamide gradient gels supplied by Thermo Fisher Scientific and employed according to the manufacturer's instructions ((3); Fig. S1A, B and C). Fractions containing the ATP synthase were pooled (Fig. S1C) and concentrated to *ca*. 500 µl by centrifugation through a Viva-spin device (molecular weight cut-off 100 kDa). The concentrated sample was applied to a Superose 6 Increase column (300 x 10 mm i.d.; GE Healthcare) equilibrated in buffer consisting of 20 mM Tris, pH 7.4, 150 mM NaCl, 2 mM ATP, 2 mM MgSO4, 10% (w/v) glycerol, 0.05% (w/v) Brij-35 and 0.1 mg/ml of a solution containing POPC, POPE, POPG and CL (3:1:1:1, by wt) at a flow rate of 0.25 ml/min (Fig. S1D). Fractions of 250 µl were collected. For samples to be used for cryo-em, the buffer was supplemented with 2 mM EDTA (unless otherwise stated) and glycerol was omitted. Fractions were analysed by SDS-PAGE and BN-PAGE (see Fig. S1E and F). Samples were applied to a glow discharged 400 mesh copper supported continuous carbon film and negatively stained with 2% (w/v) uranyl acetate. The oligomeric states of the complexes were examined in a Tecnai 12 transmission electron microscope operated at an accelerating voltage of 120 kV, at 30,000 x nominal magnification, and a defocus of -2.5 µm (Fig. S1, G, H and I). The purification is summarised in Fig. S1 of the Supplementary Results section.

Preparation of grids for cryo-em. Both sides of UltrAUFoil (R0.6/1.0 or R1.2/1.3 mesh) TEM supports were glow discharged at 25 mA under vacuum for 30 sec. Then the supports were put into tubes (0.5 ml) containing a small portion of an ethanolic solution of 5 mM mercaptopoly(ethyleneglycol)carboxylic acid (PEG-thiol) (4). The tubes were flushed with gaseous N_2, sealed and stored in a secondary N_2 flushed container for at least 48 h. Then the grids were washed three times with absolute ethanol and dried in air. A sample (3 µl) of purified dimeric ATP synthase (protein concentration 4.5 mg/ml) was applied to the grid inside the environment chamber of a Thermo Fisher Vitrobot MkIV maintained at 21ºC and 100% relative humidity. The sample was allowed to penetrate through the holey support and to distribute to both sides of the grid surface for *ca.* 15 sec. Then the grids were blotted with filter paper for 8-10 sec and vitrified in liquid ethane.

Data collection. High-resolution data sets were collected with two Titan Krios cryo-electron microscopes (FEI, Field Electron and Ion Company, now ThermoFisher Scientific), one at the Department of Biochemistry, the University of Cambridge, U.K. (UoC), and the other at eBIC (electron bio-imaging centre) at the Diamond Light Source, Harwell Campus, Oxford, U.K. The microscopes were operated at an accelerating voltage of 300 kV, and were equipped with Gatan K2 Quantum detectors operated in electron counting mode at a nominal sampling rate of 1.048 or

1.07 Å/pixel. Gain normalised images were acquired with the Gatan K2 Quantum camera operated with a post-column imaging energy filter set to a slit width of 20 eV at a dose rate of *ca.* 4.6 electrons/$Å^2$/s, for a total exposure time of 12 sec and a total dose of approximately 55 electrons/$Å^2$ fractionated over 40 individual frames. The images were collected with a defocus range of -0.9 to -2.7 µm, with an autofocussing routine every 10 µm. During automated data collection, the microscope was controlled with EPU (ThermoFisher). Two data-sets of 2,238 and 4,096 dose-fractionated movies were collected at UoC, and a third of 4,267 movies at eBIC. Representative cryo-em images are shown in Fig. S2A-C).

Structure determination of dimeric ATP synthase. Image processing procedures are described in detail in Schemes S1-S3. Briefly, the three data sets were merged, and structures of the monomeric enzyme were produced (Scheme S1). The resolution of the reconstructions was increased by exploiting the pseudo-c2 symmetry of the dimer (Scheme S1). Then the quality of the map and the resolution of specific regions were improved by focussed refinement of the stator, PS and membrane domain (see Scheme S2). Fourier shell correlation curves and local resolution estimations were calculated using RELION and are detailed in Scheme 3 (5–7).

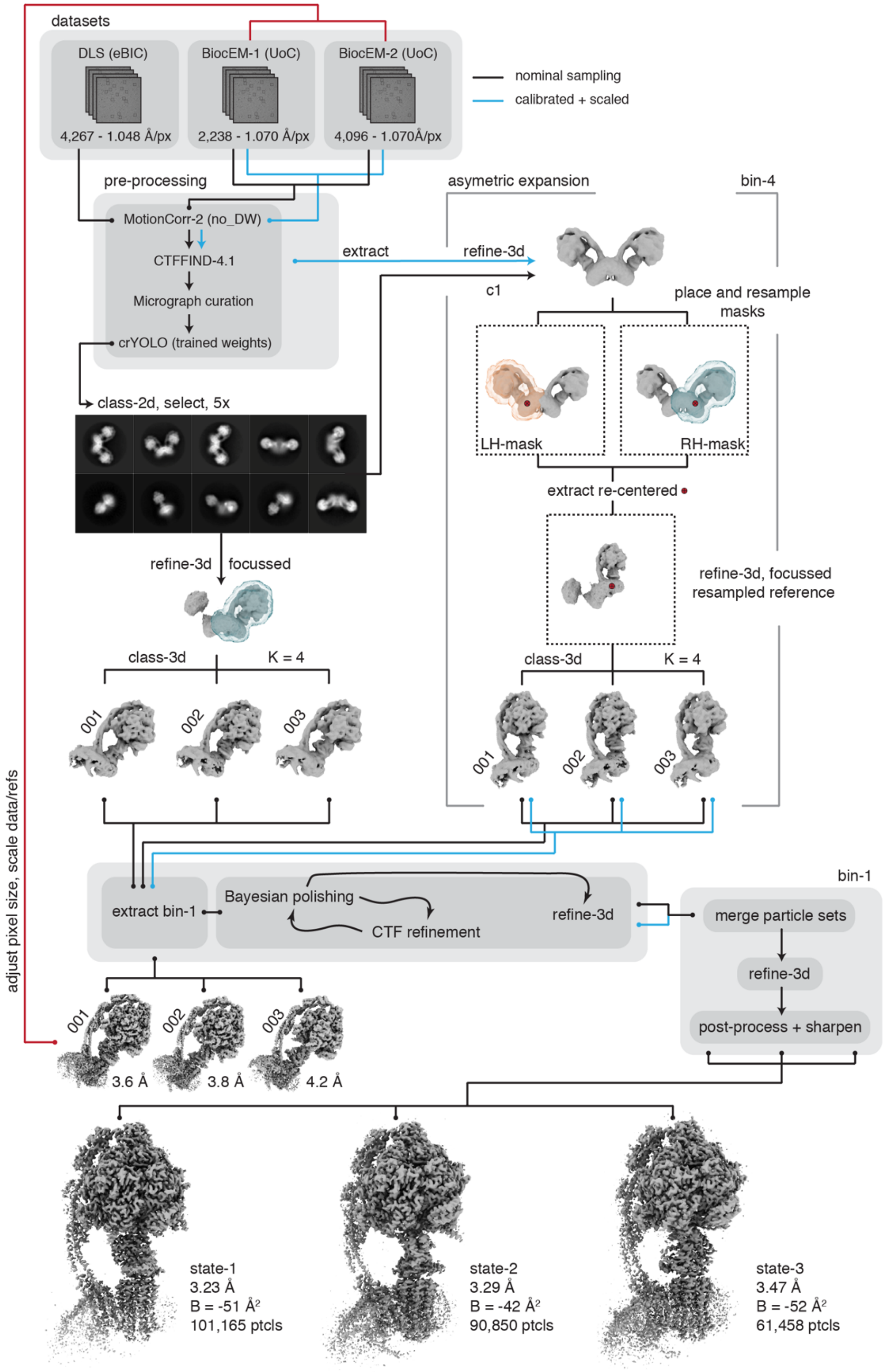

Scheme S1. Data processing of single particle images of dimeric bovine ATP synthase leading to a high-resolution reconstruction of the monomeric enzyme in catalytic states 1, 2 and 3. Images from the three datasets (4,267, 2,238 and 4,096 exposures) were processed independently at their nominal sampling rates (black lines, left process chain), and then calibrated with bovine F_1-ATPase inhibited with residues 1-60 of IF_1 (PDB 2V7Q). First, beam-induced motion and stage drift in the gain-normalised movie frames were corrected with MotionCorr2 (8) without

dose weighting, and initial CTF parameters were determined from the motion corrected frame sums with CTFFIND4.1 (9). Images displaying significant drift, or where Thon rings were not fitted reliably below 7.5 Å, were removed by visual inspection and curation, as were others with non-vitreous ice and contaminants. Automated particle picking was carried out with the neural network-based particle picker crYOLO (10). Particles were extracted at a down-sampled pixel size of sampling rate x 4 and subjected to several rounds of reference free 2D-classification and subset selection in RELION (6, 7, 11, 12). A selection of representative 2D averages is shown. This particle set was refined against a map of the bovine ATP synthase dimer prepared from an earlier unpublished data-set to produce an updated reference. Then particles were refined against the updated reference in the presence of a soft monomer shaped mask (blue). These particles were classified without particle re-alignment using the consensus orientations determined by the previous refinement, with the monomer mask in place, in order to separate particles by rotational state defined by the position of the protruding density of monomeric IF_1 relative to the PS, and by the asymmetry of the central stalk. These reconstructions are labelled 001, 002 and 003 on the left. Particles not classifying into any of the three rotational states were discarded (10,198, 3,216 and 8,163 particles from the DLS, BiocEM-1 and BiocEM-2 datasets, respectively. See Scheme1). Then the classified particle subsets were re-extracted at the nominal sampling rate in a box of 500 pixels, refined with the monomer mask in place, and subjected to Bayesian particle polishing and iterative rounds of CTF parameter refinement in RELION. The final maps were post-processed (5), sharpened and compared to PDB 2V7Q (13) to determine the calibrated sampling rate (red line). The resolutions of the maps at this stage were *ca.* 3.6-4.2 Å. The calibrated sampling rates of the eBIC and UoC data-sets were both 1.048 Å/pixel. If required, the data were corrected for beam induced motion at the correct pixel size and CTF parameters were re-calculated (blue lines). Then the curated particle sets from each data-set were prepared for asymmetric expansion (black and blue lines, right process chain), to exploit the pseudo-c2-symmetry of the dimeric complex. First, particle sets were extracted, downscaled to sampling rate x 4 and the consensus dimer refinement was repeated without the imposition of c2 symmetry to generate orientation data. A mask encompassing the membrane domain of one monomer in the dimeric pair was resampled onto each monomer in the reference produced by this refinement. The centers-of-mass of the resampled masks were calculated and subsequently used to extract new particle images centred on the right and left monomers of each molecule (defined here with respect to the symmetry axis of the molecule, viewed perpendicular to the x axis of the volume) according to the orientations determined for each particle by the previous consensus refinement. The new particle centres are indicated approximately by red circles. This procedure increased the number of particles available for refinement of a monomeric structure. Thus, these particle sets were combined and refined against a rotated and translated dimer reference in which the membrane domain of the right monomer was situated in the centre of the volume, with the central axis of the F_1-c_8 sub-complex aligned to the z-axis. This refinement was performed with a monomer mask, similarly resampled in the centre of the volume. Particles were classified without particle re-alignment with the consensus orientations determined by the previous refinement, whilst employing the monomer mask, to separate particles into their rotational states. Then the curated and sorted particle coordinates were re-extracted from the movie sums at a sampling rate of 1.048 Å/pixel in 500 pixel boxes, and subsets representing rotational states 1, 2 and 3 of the monomer were refined, as above for the down-sampled images. Per-particle CTF parameters were determined with RELION-3.1, and particles, with their associated per-particle defocus estimates, were subjected to Bayesian polishing and subsequent rounds of refinement and per-particle defocus estimation, with a final iteration including beam-tilt estimation (12, 14). Higher-order aberrations were not corrected. The final unfiltered half-maps were post-processed in RELION to estimate the resolution (based on the FSC0.143 criterion), filter them accordingly and to automatically estimate and apply a sharpening B-factor (5). They are shown at the bottom of the panel. Additional unsharpened maps or maps with ad hoc B-factors were generated to aid in model building and interpretation of density. The final resolutions for states 1-3 were 3.23, 3.29 and 3.47 Å, respectively, with applied B-factors of -51, -42 and -52 $Å^2$, respectively (see Scheme 3).

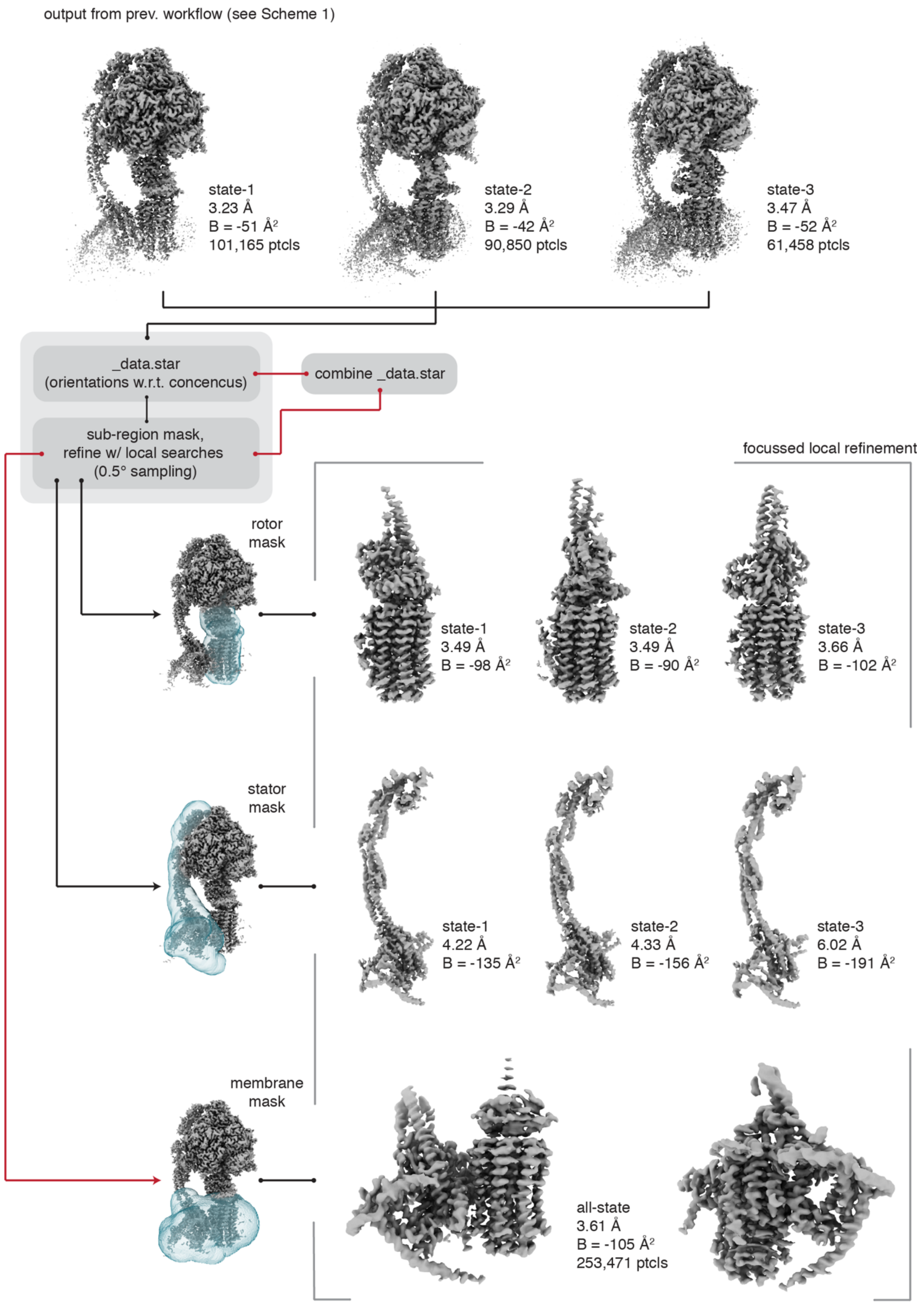

Scheme S2. Local refinement of sub-regions of bovine ATP synthase. Masks (blue) were created for the rotor (the central stalk and the c_8-ring), the stator (the PS subunits OSCP, b, d and F_6, plus membrane associated subunits a, A6L, e, f, g, j and k), and the membrane domain (the c_8-

ring and subunit a, the membrane domain of subunit b, plus associated subunits A6L, e, f, g, j and k). These masks were employed during refinements, initialised from the previously determined consensus orientations, with restricted angular and translation searches of 0.5° and 3 pixels, respectively. Local refinements of the rotor and the PS were carried out on individual particle subsets representing each rotational state (black lines), whilst the local refinement of the membrane domain was performed by combining all particle sets (red lines). Whilst this map displays some heterogenous density in the c_8-ring, novel features such as the presence of lipid molecules became apparent upon refinement of the merged particle set. Although the estimated resolutions of these maps are lower than that of the corresponding consensus monomer structures, the quality of the density had improved significantly. Therefore, regions of subunits could be assigned based on their sequences, with increased confidence during model building. Also, novel features such as the lipids emerged, and the structure of regions of γ-subunits (residues 62-66 and 97-100) that were disordered in crystal structures were resolved.

Scheme S3. Fourier shell correlations and estimates of local resolution of reconstructions of bovine ATP synthase. Half-maps were masked with soft-edge masks with cosine edges and the resolutions of the reconstructions were estimated in RELION. The effect of the mask was deconvoluted by a phase-randomisation procedure (5). The corrected Fourier-shell correlations are shown on the left as a function of spatial frequency. A suitable sharpening factor was estimated from automatic B-factors determined in RELION. Local resolutions (see rainbows above) were derived in RELION by iterating the same post-processing routine on small spherical sub-regions of the unfiltered half-maps (12).

Model building and refinement. Model building into focussed maps was performed with COOT (15) and real space refinement with PHENIX (16–18). Lipids were modelled into density with ISOLDE (19). The starting model comprised the following crystal structures of sub-domains of bovine ATP synthase: F_1-ATPase inhibited with residues 1-60 of IF_1 (PDB:2V7Q) (13), a PS fragment (PDB:2CLY) (20), the c_8-ring (PDB:2XND) (21) and the OSCP subunit from the structure of bovine F_1-ATPase complexed with the membrane extrinsic region of the PS (PDB:2WSS) (22). Subunit a was based on the cryo-em structure of bovine ATP synthase at 7 Å resolution (PDB:5ARA) (23). Model geometry and density fit validation was performed by MolProbity (24, 25) and EMRinger (26), respectively.

Analysis of subunit sequences. Pairwise comparisons of the sequences of orthologous subunits of bovine, porcine and yeast ATP synthases subunits were made with CLUSTAL O (27) on www.uniprot.org, and their secondary structures were predicted with PSIPRED (28, 29).

Figures, movies and animations.
Figures and movies were prepared using USCF ChimeraX (30).

Movie 1. Each ATP synthase subunit was extracted from the cryo-em density of the corresponding focussed locally refined reconstruction and displayed one by one. The positions of the monomers were defined by the reconstruction of the state 1: state1 dimer which we have described elsewhere.

Movie 2. The composite atomic models of the bovine ATP synthase monomer in rotational states 1, 2 and 3 were aligned to the a-subunit of the state-1 model and coordinate trajectories were calculated from state1 to state 2, state 2 to state 3 and state 3 to state 1, completing one full rotation of the enzyme. The trajectories are repeated in order.

Movie 3. The atomic models of the OSCP, coloured teal, from rotational states 1, 2 and 3 of the bovine ATP synthase monomer were aligned to residues 116-188 of the OSCP in the state 1 model

and coordinate trajectories were calculated from state 1 to state 2, state 2 to state 3 and state 3 to state 1.

Supplementary results

Purification of dimeric bovine ATP synthase.

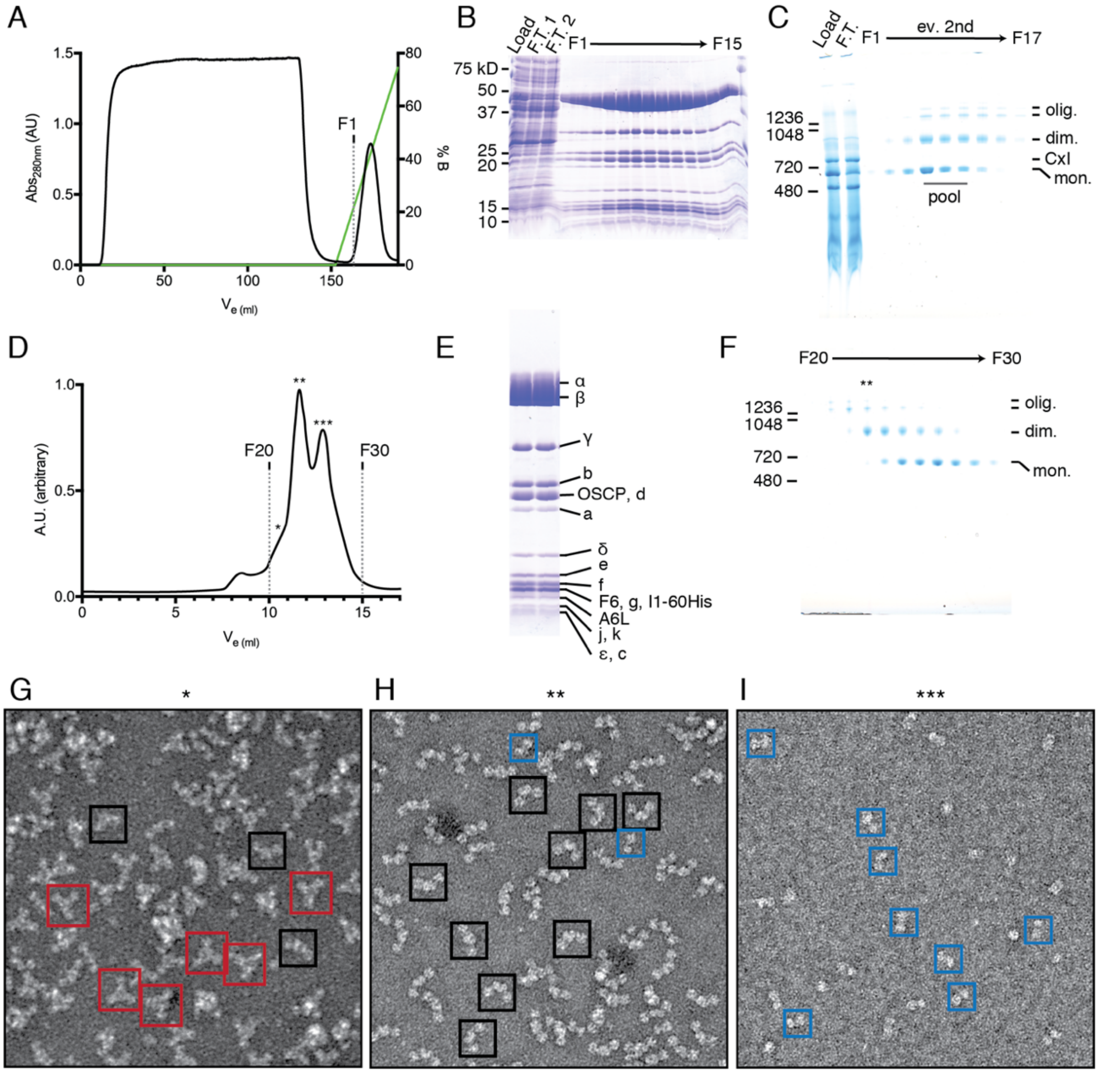

Fig. S1. Purification of dimeric bovine ATP synthase. The enzyme was extracted from mitochondrial membranes in the presence of digitonin and DDM, and inhibited by residues 1-60 of bovine IF_1 with a His_6 C-terminal tag. *A*, affinity chromatography on a Ni-Sepharose column (5 ml) equilibrated in buffer containing GDN (0.1%) and exogenous phospholipids (POPC, POPE, POPG and cardiolipin) of a sample of extracted membranes (120 ml; protein concentration 8.5 mg/ml). The proteins were eluted with an imidazole gradient from 25-500 mM (green), collecting 2 ml fractions and the uv absorbance of the eluate was monitored at 280 nm (black). The elution volume of fraction 1 is indicated by the grey dashed line. For additional details, see Supplementary Materials and Methods. *B*, analysis by SDS-PAGE of fractions in part A. The migration positions of molecular weight markers are indicated in kDa on the left. *C*, analysis of fractions by BN-PAGE. The migration positions of molecular weight standards are indicated in kDa on the left, and those of monomers, dimers and higher oligomers on the right. *D*, size exclusion profile (black trace) of the concentrated pooled fractions indicated in *C* on a column of Superose 6 Increase at a flow rate of 0.25 ml/min in the presence of Brij-35 and phospholipids. *E*, analysis by SDS-PAGE of *ca.* 10-

12 µg of fractions, indicated by double asterisks in *D,* from the size exclusion column. The migration positions of subunits of bovine ATP synthase are indicated on the right. Part *F*, analysis of fractions indicated in *D* by BN-PAGE. *G-I,* analysis by electron microscopy of negatively stained of samples from fractions 21, 23 and 28 respectively, indicated by asterisks in *D*. Red, black and blue boxes contain trimeric, dimeric and monomeric assemblies, respectively. Samples from fraction 23, indicated by two asterisks in *D* and *F*, were examined by cryo-em.

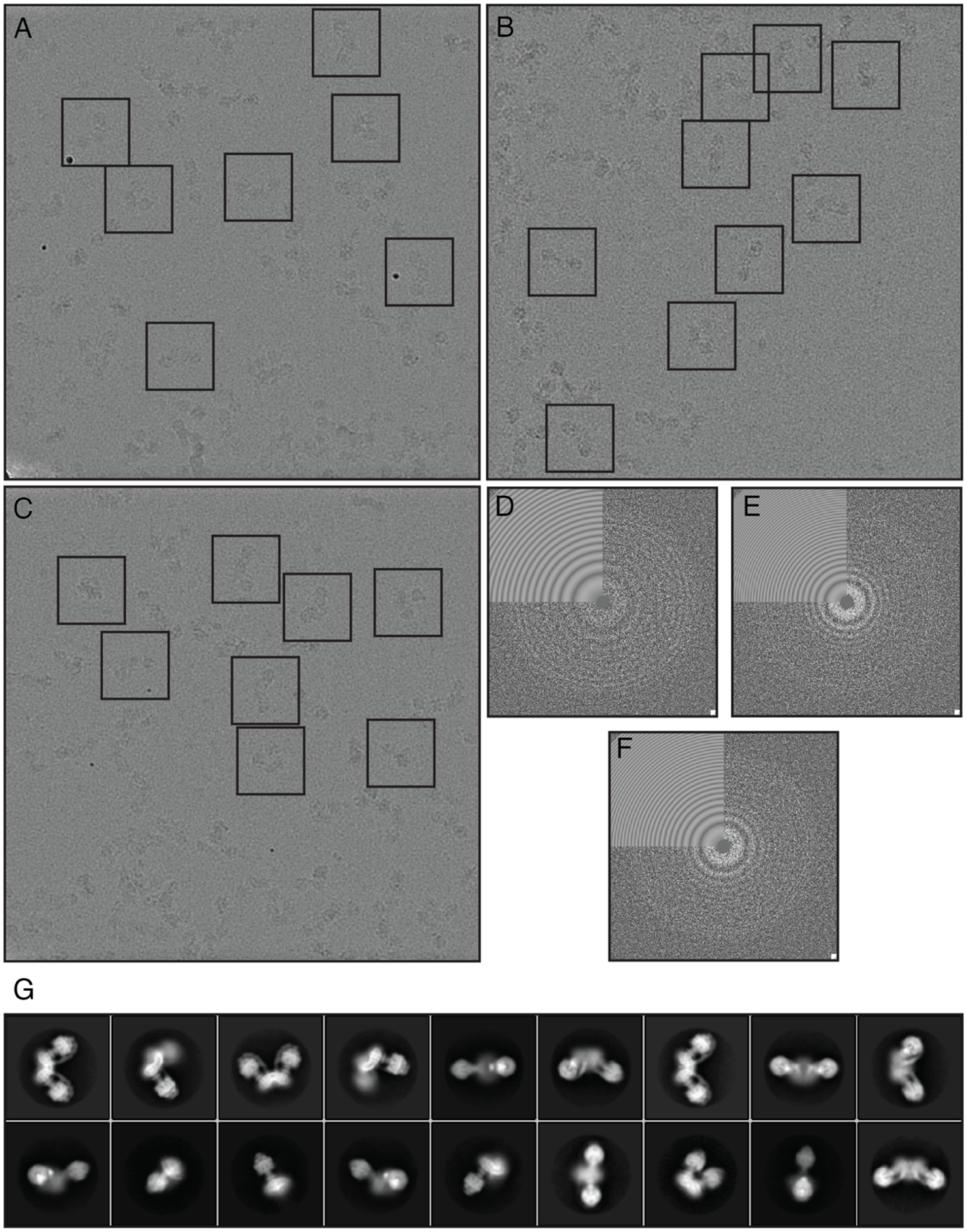

Fig. S2. Representative cryo-em images of dimeric bovine ATP synthase. *A*-*C*, dimeric samples, with examples of dimeric particles in boxes. *D*-*F*, representative radial power spectra of micrographs collected in *D* at low, in *E* at high, and in F intermediate, defocus values with Thon rings visible to *ca.* 4 Å. In most cases, they were fitted accurately to 3.3 Å by CTFFIND-4.1 (9). *G*, representative 2D-class averages in various orientations in the vitreous ice layer calculated at a

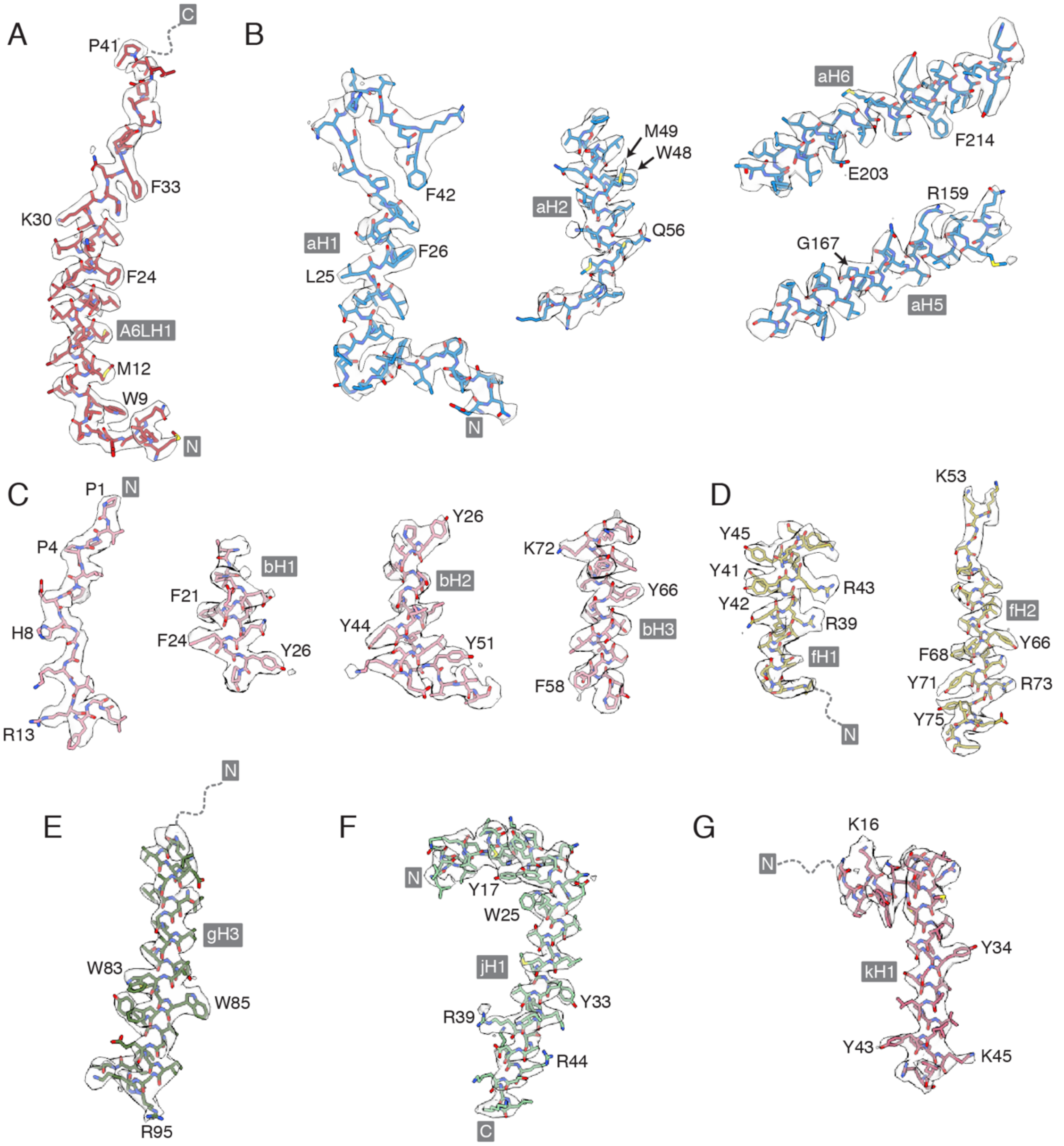

Fig. S3. Examples of cryo-em densities extracted from the focussed local refinement of the membrane domain of bovine ATP synthase. *A-G*, portions of the cryo-em densities for subunits A6L (brick red), a (cornflower blue), b (light pink), f (straw yellow), g (forest green), j (sea-foam green) and k (dark pink) respectively. The atomic model of each subunit is shown, with examples of density features facilitating assignment and sequence registration as indicated. Regions of density were extracted within 2 Å of the fitted atomic model.

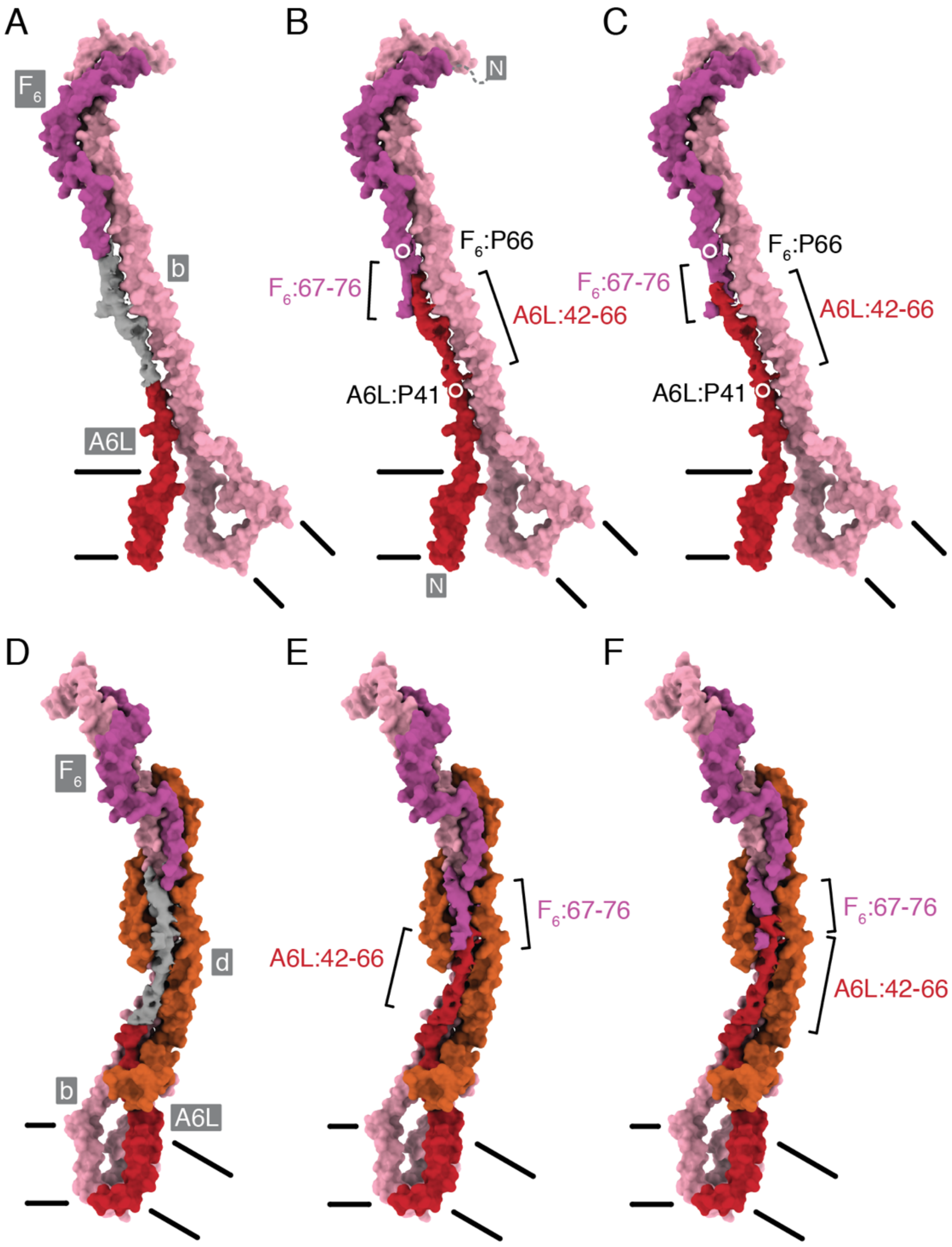

Fig. S4. A structurally ambiguous region of the bovine peripheral stalk. A region of density close to the mid-point of the bovine PS was insufficiently resolved to allow it to be modelled, and the same region was also unresolved in the porcine enzyme. The yeast PS has a different structure in this region. The density in the bovine PS corresponds to the C-terminal regions of subunits A6L (residues 42-66) and F_6 (residues 67-76) which interact. Both subunits are predicted to have extended C-terminal strands. *A*, the unmodelled density (grey) with the modelled A6L (red), and F_6 (purple) subunits, and the b-subunit (pink) shown for reference; *B* and *C*, two alternative interpretations, in *B*, with F_6 on the outside, and in *C*, with A6L on the outside. White circles indicate the last residues able to be built in each subunit, residue A6L-Pro-41 and residue F_6Pro-66. In *A-C*, subunit d was omitted for clarity. *D-F*, as above but with the view is rotated 90° from left to right with the d-subunit (orange) added to complete the interactions in this region of the PS.

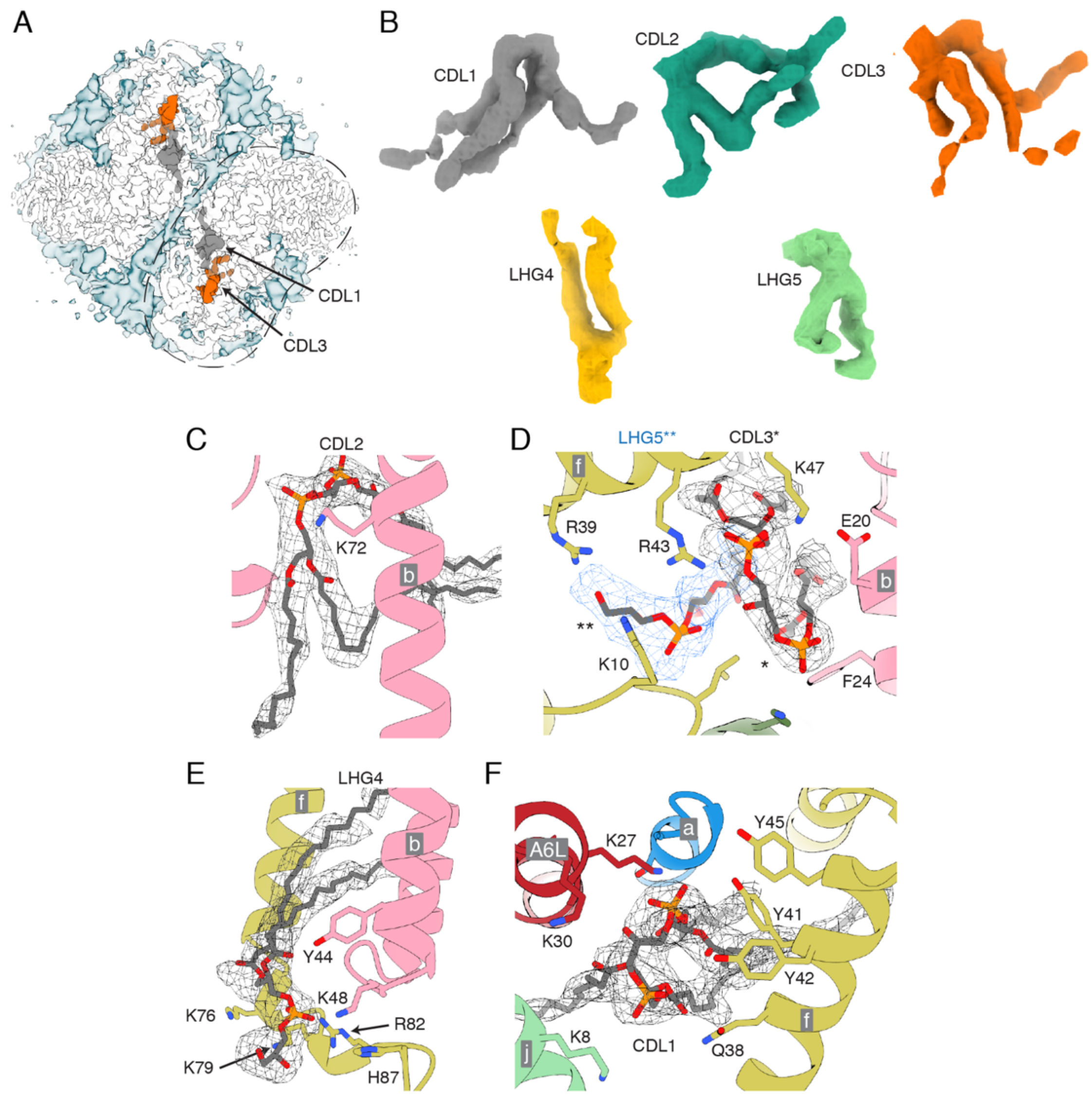

Fig. S5. Bound lipids resolved in the membrane domain of bovine ATP synthase. *A*, view from the matrix side of the IMM of the dimeric membrane domain with the PS and catalytic domain removed for clarity. Protein density is in grey transparency, density from an intact dimer reconstruction representing the detergent micelle is in blue transparency and the densities for CDL1 and CDL3 are dark grey and orange, respectively. Note the proximity of the CDL1 molecules to the monomer-monomer interface; *B*, cryo-em densities of lipids in the membrane domain with CDL1, CDL2, CDL3, LHG4 and LHG5 in dark grey, turquoise, orange, yellow and light green, respectively; *C*-*F*, interactions of CDL2, CDL3 and LHG5, LHG4 and CDL1 with nearby polar residues. Subunits b, f, a, A6L and j are light pink, straw yellow, cornflower blue, brick red and sea-foam green, respectively. The alkyl chains of CDL1 deviate significantly from the perpendicular axis of the membrane. One chain runs beneath the amphipathic α-helix of subunit j traversing directly toward the second monomer, and a second fills a non-protein void beneath fH1, together with CDL3 and LHG5. CDL2 and LHG4 line the back of the proton inlet channel.

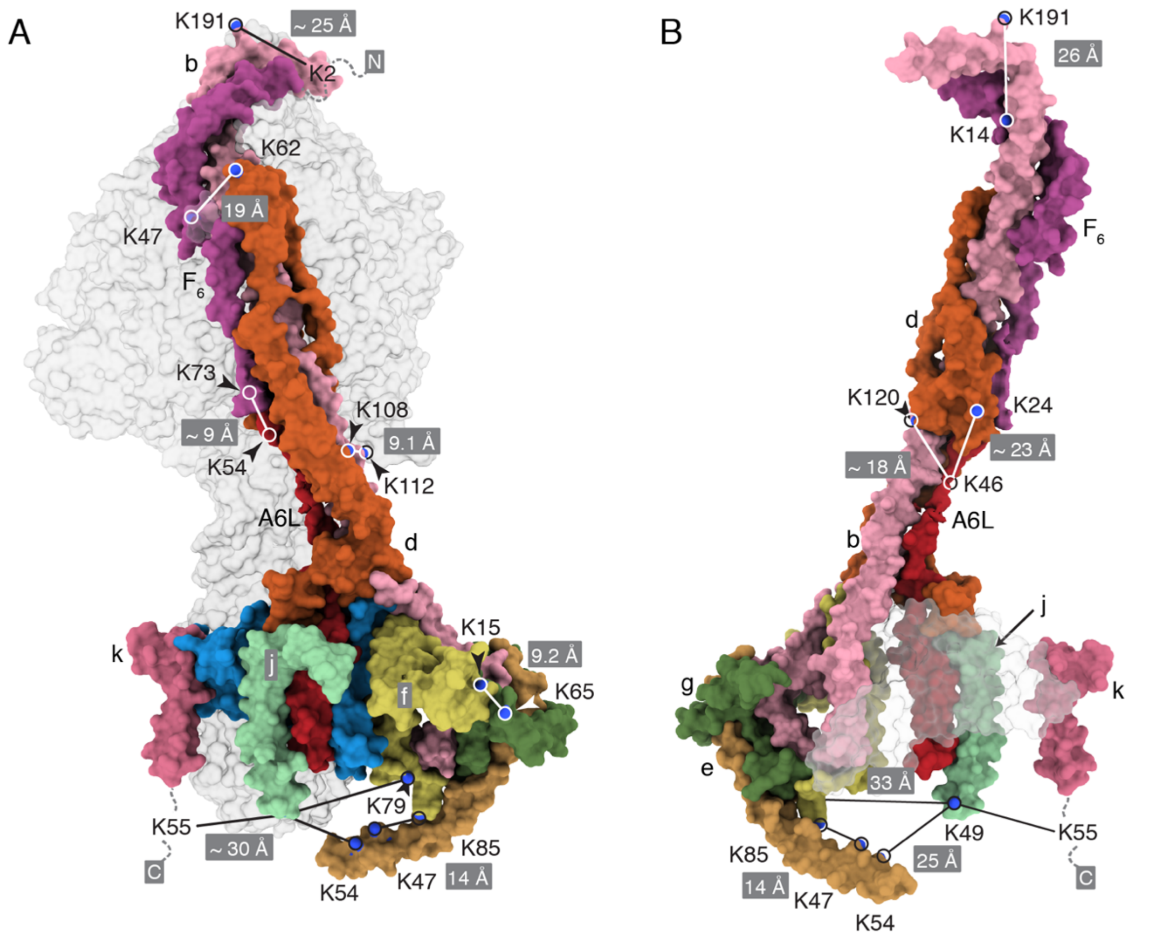

Fig. S6. Compatibility of the structure of bovine ATP synthase with chemical cross-links introduced into the membrane and PS. PS subunits b, d and F_6 are light pink, orange and magenta, respectively, and membrane subunits a, A6L, e, f, g, j and k are cornflower blue, brick red, khaki, straw yellow, forest green, sea-foam green and dark pink, respectively. *A* and *B*, the membrane domain and PS of the bovine ATP synthase monomer, with the catalytic domain and rotor in grey transparency, viewed in *A* from approximately between monomers in the dimeric enzyme and in *B*, rotated by 180°, with the catalytic domain and rotor removed for clarity, and the a-subunit in grey transparency. Where the C-termini of subunits F_6 and A6L meet in the middle of the PS, the surface representation of subunits F_6 and A6L has been extended with cryo-em densities that indicate their extents, but they were not of sufficient quality to allow the sequence to be modelled (see Fig. S4). The measured distances in Å between lysine residues found earlier to be cross-linked with disuccinimidyl-suberate in a preparation of the monomeric enzyme (31) are indicated. The maximum inter-Cα distance between connected lysines is about 28 Å. Where cross-links to subunits k, F_6 and A6L were not fully resolved and therefore were not modelled in the atomic structure, the distances are approximate.

Reinterpretation of the structure of dimeric porcine ATP synthase.

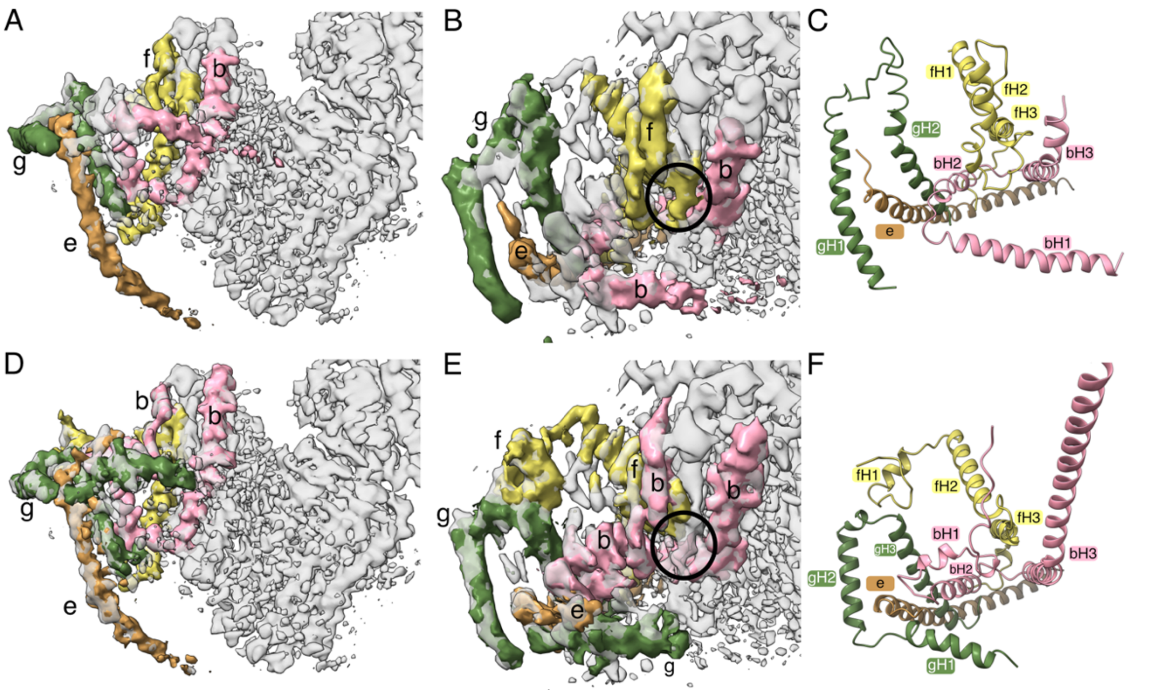

Fig. S7. Re-interpretation of the membrane domain of porcine ATP synthase. The porcine density (grey) is deposited at EMD-0668 (32). *A* and *B*, respectively, views of the same region of the membrane domain of porcine state E from the side and rotated towards the viewer by 90°, with their attributed subunits; *C*, deposited porcine subunits b, e, f, and g in cartoon representation viewed as in *B*; *D* and *E*, views of the same region of the porcine ATP synthase following re-interpretation of the density and re-assignment of subunits according to the bovine structure; in *D* and *E*, black circles indicate a region modelled previously as porcine subunit f, and occupied by cardiolipin CDL2 in the bovine enzyme. *F*, bovine subunits b, e, f and g in cartoon representation viewed as in *E*.

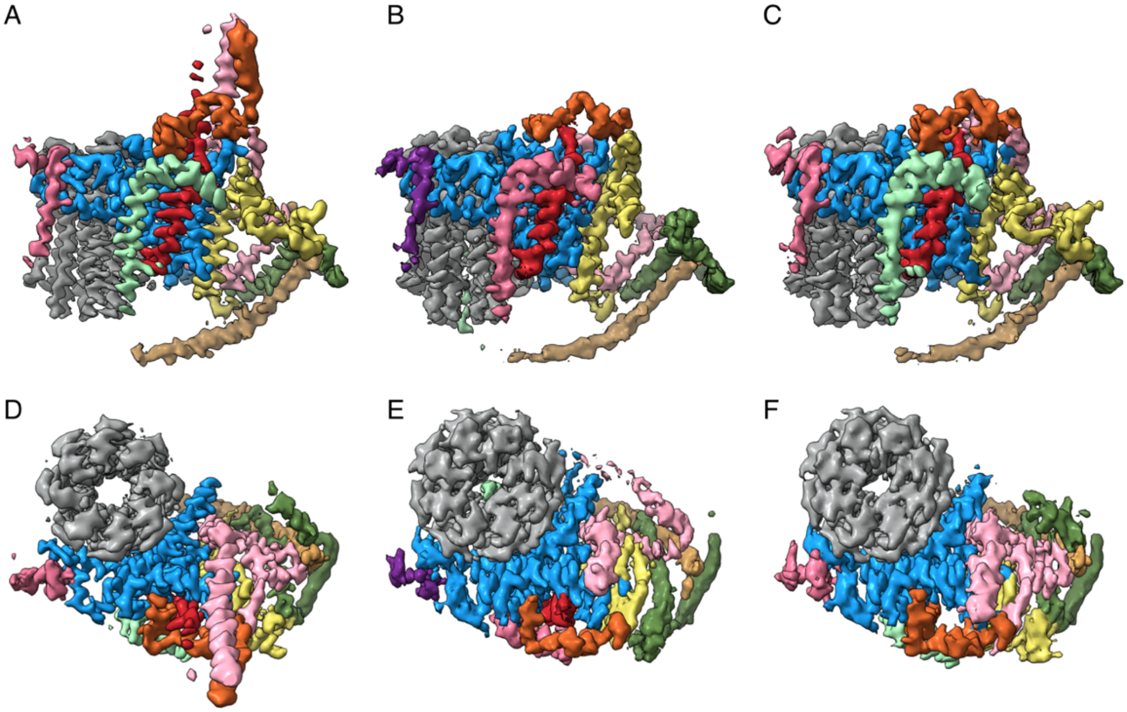

Fig. S8. Re-assignment of subunits j and k, and regions of subunits b, f and g in the membrane domain of porcine ATP synthase. Side views of *A*, the bovine enzyme; *B*, deposited porcine structure (32); *C*, re-interpreted porcine structure; *D*-*F*, orthogonal views in the plane of the membrane from the mitochondrial matrix of *A*-*C*, respectively. Subunits are coloured as follows: a, cornflower blue; b, pink; c, grey; d, orange; e, sand; f, yellow; g, dark green; A6L, red; j, pale green; and k, dark pink. In *B* and *E*, a proposed hypothetical subunit is purple.

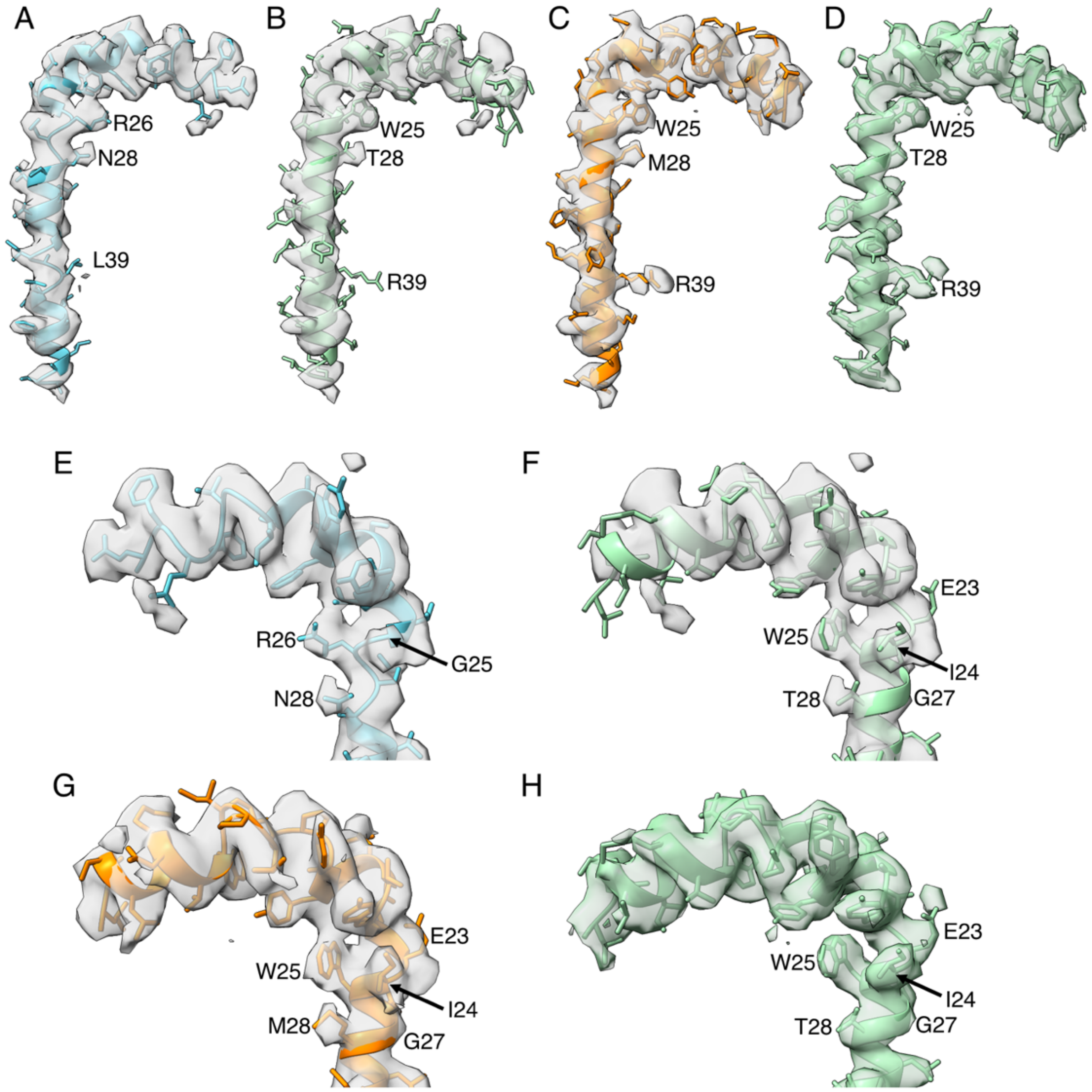

Fig. S9. Reassignment of porcine subunit j. In the porcine structure, the subunit identified as j in the structures of bovine and yeast ATP synthase was assigned incorrectly as subunit k, and porcine subunit j was placed incorrectly in the internal cavity of the c_8-ring and built as a poly-alanine α-helix (32). The sequences of the bovine and porcine j-subunits are 88.3% identical (Fig. S10). *A*, deposited porcine density and model; *B*, deposited porcine density with bovine model; *C*, porcine density interpreted with sequence of porcine subunit j and re-extracted; *D*, bovine density and bovine model; *E*-*H*, magnified views of the upper parts of *A*-*D*, respectively, rotated through 180° relative to *A*-*C*. In *C* (and *G*) and *D* (and *H*), note for example, that the substitution M28T is reflected in the difference in density at these positions.

```
j-subunit 88.3% identity

P14790|j_BOVIN   MLQSLIKKVWIPMKPYYTQAYQEIWVGTGLMAYIVYKIRSADKRSKALKASSAAPAHGHH 60
A0A4X1TX70|j_PIG MLQSLIKNVWIPLKPYYTQVYQEIWVGMGLMGFIVYKIRSADKRSKALKASSPAPAHGHH 60
                 *******:****:******.******* ***.:******************* *******


j-subunit 14.9% identity

P14790|j_BOVIN   MLQSLIKKVWIPMKPYYTQAYQEIWVGTGLMAYIVYKIRSADKRSKALKASSAAPAH--G 58
P81450|j_YEAST   MLK----RFPTPILK----VYWPFFVAGAAVYYGMSKAADLSSNTKEFINDPRNPRFAKG 52
                 **:    :.  *:      .*  ::*. . : * : *  . ...:* :  .   * .  *

P14790|j_BOVIN   HH----- 60
P81450|j_YEAST   GKFVEVD 59
```

Fig. S10. Comparison of the sequences of j-subunits of bovine, porcine and yeast ATP synthases. In the bovine enzyme, the subunit is sometimes referred to as 6.8PL (6.8 kD proteolipid). Above, bovine versus porcine; below, bovine versus yeast.

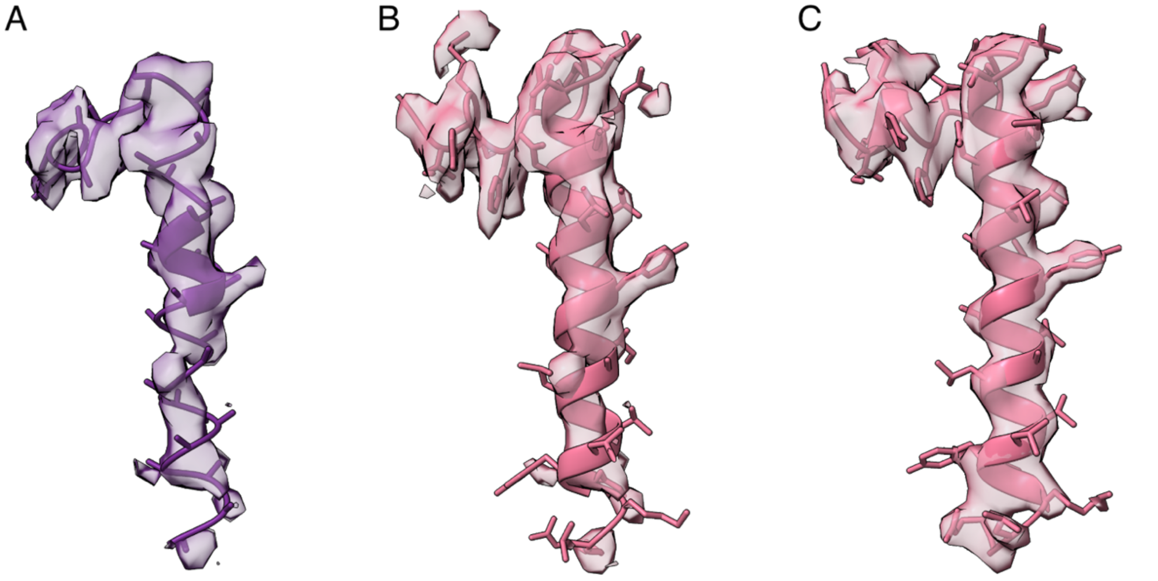

Fig. S11. Reassignment of porcine subunit k. The subunit identified as k in the bovine and yeast structures was assigned as an unidentified hypothetical protein in the porcine structure with no corroborative data. The sequences of the bovine and porcine j-subunits are 93.1% identical (Fig. S12). *A*, deposited porcine density modelled as poly-alanine; *B*, porcine density re-extracted after assignment of the sequence of porcine subunit k; *C*, density and model of bovine subunit k.

```
k-subunit 93.1% identity

Q3ZBI7|k_BOVIN AGPEADAQFHFTGIKKYFNSYTLTGRMNCVLATYGSIALIVLYFKLRSKKTPAVKAT 57
F1RFD4|k_PIG   AGPETDAQFQFTGIKKYFNSYTLTGRMNCVLATYGGIALLVLYFKLRSKKTPAVKAT 57
               ****:****:*************************.***:*****************


k-subunit 13.6% identity

Q3ZBI7|k_BOVIN AGPEADAQFHFTGIKKYFNSYTLTGRMNCVLATYGSIALIVLYFKLRSKKTPAVKAT--- 57
P81451|k_YEAST ----MGAAYHFMGKAIPP--------HQLAIGTLGLLGLLVVPNPFKSAKPKTVDIKTDN 48
                    .* :** *              : .:.* * :.*:*:   ::* *  :*. .

Q3ZBI7|k_BOVIN --------------------
P81451|k_YEAST KDEEKFIENYLKKHSEKQDA 68
```

Fig. S12. Comparison of the sequences of k-subunits of bovine, porcine and yeast ATP synthases. In the bovine enzyme, the subunit is sometimes referred to as DAPIT (diabetes associated protein in insulin sensitive tissue). Above, bovine versus porcine; below, bovine versus yeast.

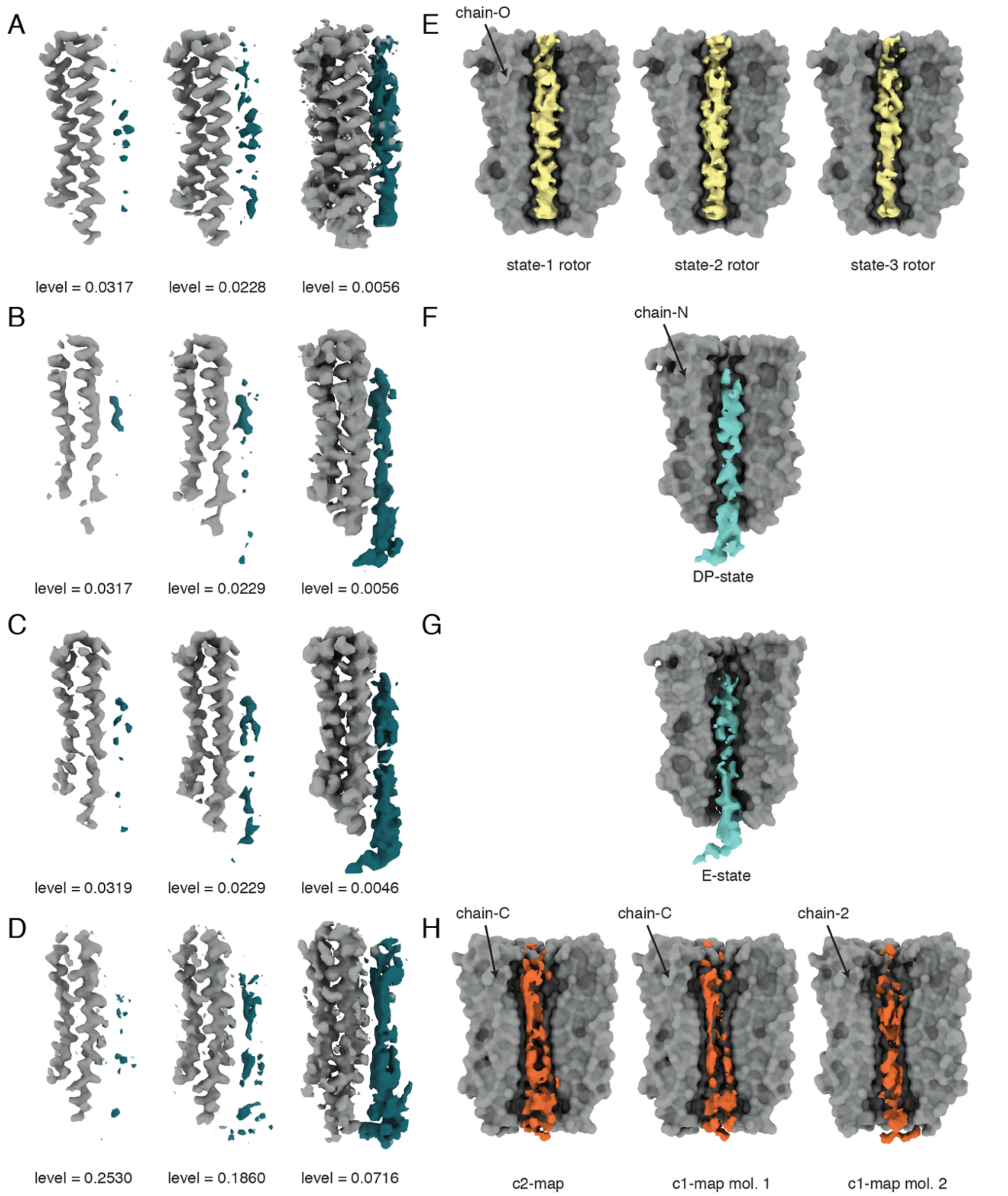

Fig. S13. The noisy, poorly defined cryo-em densities observed in the inner cavities in the c_8-rings in bovine and porcine ATP synthases and in the c_{10}-ring in the yeast enzyme. *A*-*D*, cryo-em density of a single c-subunit monomer (grey) plus the density in the inner cavity of the c-rings (teal) at various map thresholds in *A*, bovine, *B* and *C* porcine, and *D*, yeast ATP synthases; *E*, the same bovine density (yellow), extracted from each of the three reconstructions of the rotors, with the solvent excluded molecular surface of the c_8-ring. The four foremost c-subunits have been removed to reveal the inside of the cavity; *F* and *G*, the density (turquoise) from the DP and E state reconstructions, respectively, in the porcine enzyme (EMD-0670, EMD-0668) (32); *H*, densities (orange) of the dimeric membrane domain from *S. cerevisiae* extracted from the c2-symmetrised reconstruction and from each molecule of the non-symmetrised reconstruction (EMD-7036, EMD-

3037) (33). As the yeast samples lacked membrane extrinsic regions, rotational states of the c_{10}-ring were not resolved; the inner density shows signs of rotational averaging of the occupying entity.

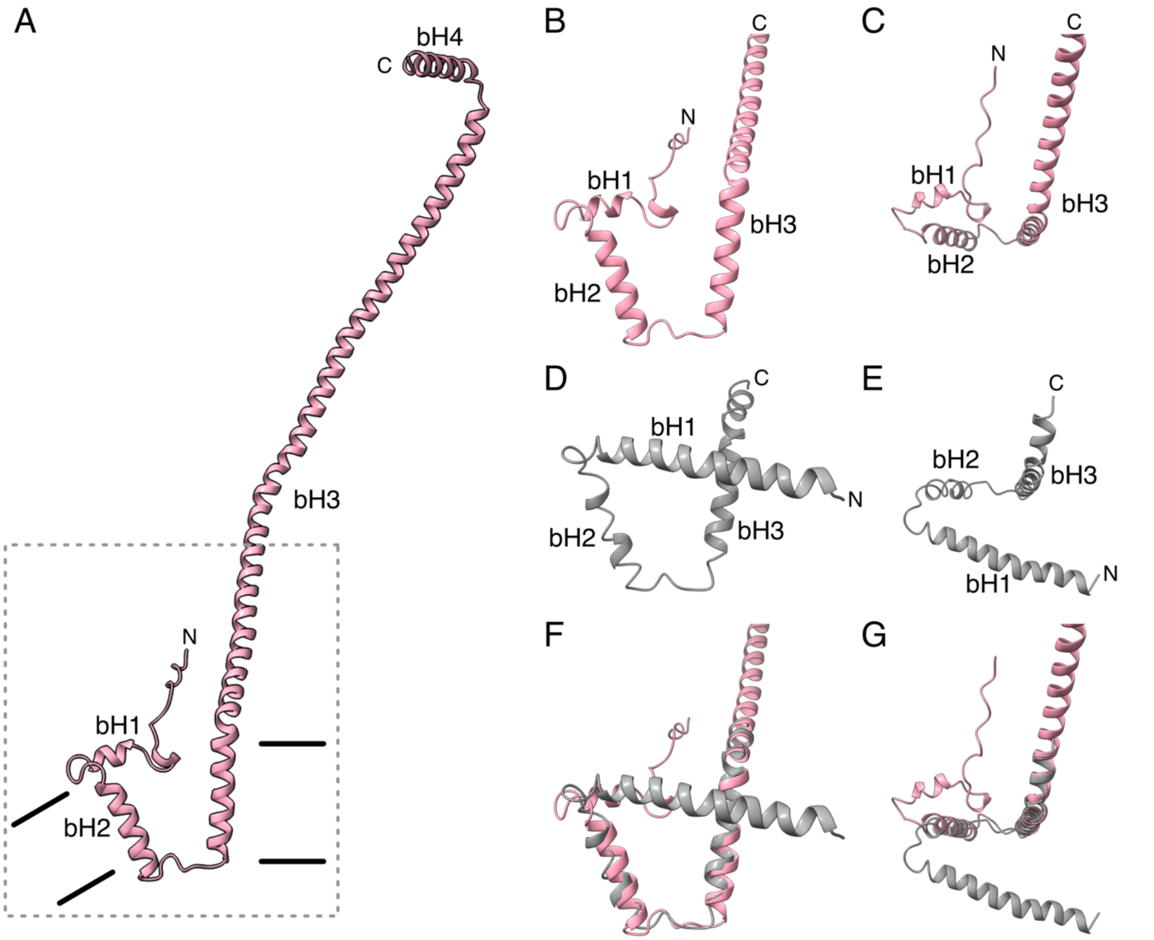

Fig. S14. Reinterpretation of the structure of porcine subunit b. In the published porcine b-subunit (32), the N-terminal region preceding bH2 was built incorrectly into density that represents α-helix gH1, based on the incorrect assumption that the structures of yeast and porcine b-subunits would have the same topography. With a sequence identity of 95.3%, the bovine subunit b model fits the porcine map and accounts for more of the unmodelled density (Figs. S7, and S8). *A*, the structure of the predominantly α-helical bovine b-subunit with an extended N-terminal region (residues 1-20), followed by amphipathic α-helix, bH1 (residues 19-28), transmembrane α-helix, bH2 (residues 32-48), an amphipathic extended section (residues 49-54), a long, mainly straight α-helix, bH3 (residues 55-186), including a transmembrane span (residues 55-73) extending in the peripheral stalk to the top of the F_1-domain, and terminated by the C-terminal α-helix, bH4 (residues 190-209), which interacts with the OSCP subunit. The N-terminal region runs parallel to bH3. The dashed box contains the region used in the following panels. *B* and *C*, top and side views, respectively, of bovine subunit b (pink); *D* and *E*, top and side views, respectively, of the published porcine b-subunit (grey); *F* and *G*, top and side views, respectively, of the superimposed bovine (pink) and porcine (grey) b-subunits. The reinterpreted porcine b-subunit resembles the bovine orthologue.

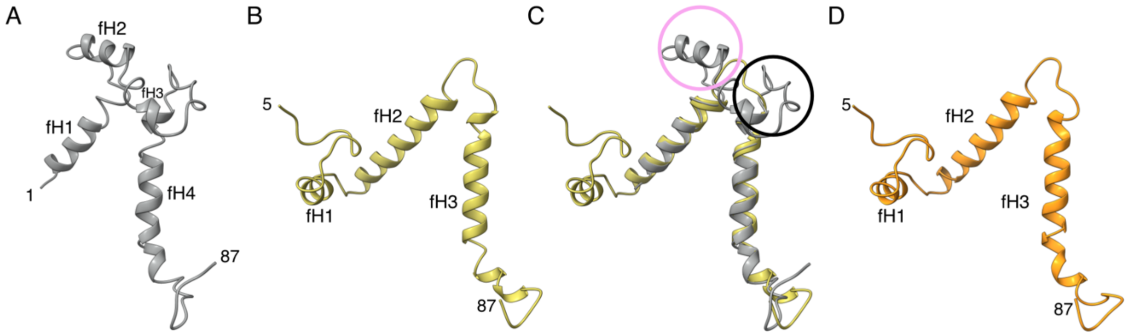

Fig S15. Reinterpretation of the structure of porcine subunit f. The sequences of the bovine and porcine f-subunits are 93% identical (Fig. S15). *A*, the deposited porcine model (32); *B*, the bovine model; *C*, the porcine and bovine models superimposed. The pink circle indicates a region in the porcine subunit built into density that in reality is the N-terminus of the b-subunit, and the black circle contains a loop introduced incorrectly into porcine subunit f before trans-membrane α-helix fH3. This region is better resolved in the bovine and yeast maps and is modelled as cardiolipin CDL2 in the bovine structure.; *D*, the re-interpreted porcine f-subunit. In the deposited porcine data, connected density (see Fig. S7 above) precedes the residue incorrectly assigned as the N-terminus, corresponding to fH1 and the preceding N-terminal strand in *B* and *D*.

```
f 93.2% identity

Q28851|f_BOVIN MASVVPLKEKKLLEVKLGELPSWILMRDFTPSGIAGAFQRGYYRYYNKYVNVKKGSIAGL 60
Q95339|f_PIG   MASVVPLKDRRLLEVKLGELPSWILMRDFTPSGIAGAFQRGYYRYYNKYVNVKKGSVAGL 60
               ********:::*********************************************:***

Q28851|f_BOVIN SMVLAAYVFLNYCRSYKELKHERLRKYH 88
Q95339|f_PIG   SMVLAAYVVFNYCRSYKELKHERLRKYH 88
               ********.:******************


f 19.6% identity

Q28851|f_BOVIN -ASVVPLKEKKLLEVKLGELPSW------ILMRDFTPSGIAGAFQ--RGYYRYYNKYVNV 51
Q06405|f_YEAST VSTLIPPKV--VSSKNIGSAPNAKRIANVVHFYKSLPQGPAPAIKANTRLARYKAKYFDG 58
                ::::* *   : . ::*. *.       : : .  *.* * *::      **  **.:

Q28851|f_BOVIN KKGSIAGLSMVLAAYVFLNYC-RSYKELKHERLRKYH 87
Q06405|f_YEAST DNASGKPLWHFALGIIAFGYSMEYYFHLRHHKGAEEH 95
               .:.*   *  .  . : :.*. . * .*:*.:  : *
```

Fig. S16. Comparison of the sequences of f-subunits of bovine, porcine and yeast ATP synthases. Above, bovine versus porcine; below, bovine versus yeast.

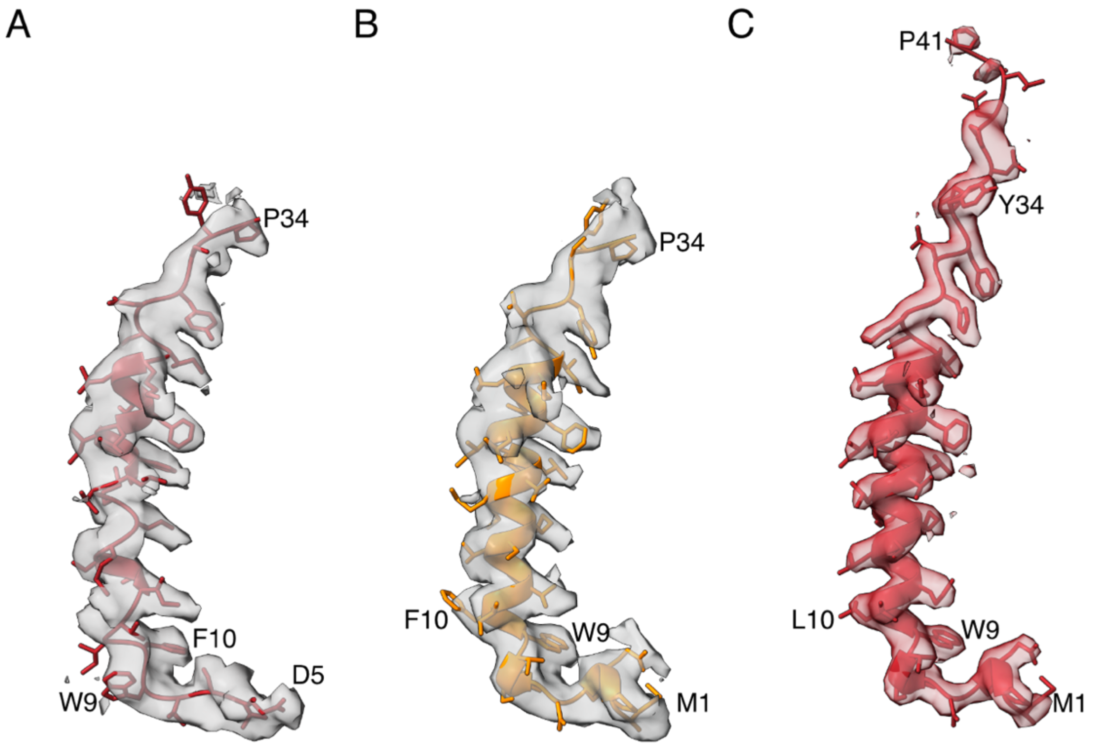

Fig S17. Reinterpretation of the structure of the porcine A6L subunit. *A*, deposited porcine model and map (32); *B*, reinterpreted porcine model in the deposited map; *C*, bovine model and map. The sequences of the porcine and bovine A6L subunits are 61.2% identical (Fig. S18). Although the porcine and bovine models are similar, porcine residues 5-20 have been built into the density incorrectly, with residue A6L-Phe-10 built into density that in reality is residue A6L-Trp-9, and with residue 20 out of position as the additional density for a π bulge was not introduced. The bovine model fits the porcine density very well. The bovine and porcine models deviate at residue 34 (Tyr in bovine and Pro in porcine subunits), and, because of masking, the porcine density does not extend further. In focussed maps of the bovine PS, the same region (residues 34-41) is poorly resolved, and residues 30-45 are not well conserved (Fig. S4, Fig. S18). However, the densities suggest that in this region the porcine and bovine subunits differ. In general, the sequence of the A6L subunit is not well conserved in mammals, and, for example, the sequences of the bovine and human subunits are only 50% identical (Fig. S18).

```
ATP8 61.2% identity

P03929|ATP8_BOVIN MPQLDTSTWLTMILSMFLTLFIIFQLKVSKHNFYHNPELTPTKMLKQNTPWETKWTKIYL 60
Q35914|ATP8_PIG   MPQLDTSTWFITITSMIMTLFILFQLKISNYSYPASPESIELKTQKHSTPWEMKWTKIYL 60
                  *********:  * **::****:****:*::.:  .**    *  *:.**** *******

P03929|ATP8_BOVIN PLLLPL- 66
Q35914|ATP8_PIG   PLLLPPR 67
                  *****

ATP8 50% identity

P03929|ATP8_BOVIN MPQLDTSTWLTMILSMFLTLFIIFQLKVSKHNFYHNPELTPTKMLKQNTPWETKWTKIYL 60
P03928|ATP8_HUMAN MPQLNTTVWPTMITPMLLTLFLITQLKMLNTNYHLPPSPKPMKMKNYNKPWEPKWTKICS 60
                  ****:*:.* ***  *:****:* ***: : *::  *. .* ** : *.*** *****

P03929|ATP8_BOVIN PLLLPL-- 66
P03928|ATP8_HUMAN LHSLPPQS 68
                     **

ATP8 15.2% identity

P03929|ATP8_BOVIN MPQLDTSTWLTMILSMF---LTLFIIFQLKVSKHNFYHNPELTPTKMLKQNTPWETKWTK 57
P00856|ATP8_YEAST MPQLVPFYFMNQLTYGFLLMITLLI-------------------------------LFSQ 29
                  ****    ::. :   *   :**:*                                :::

P03929|ATP8_BOVIN IYLPLLLPL---------- 66
P00856|ATP8_YEAST FFLPMILRLYVSRLFISKL 48
                  ::**::* *
```

Fig. S18. Comparison of the sequences of A6L (or ATP8) subunits of bovine, porcine, human and yeast ATP synthases. In the bovine enzyme, the subunit is also referred to as A6L, and in the yeast enzyme as Aap1. Above, bovine versus porcine; middle, bovine versus human; below, bovine versus yeast.

Brief descriptions of discrepancies in other subunits

Subunit a. The sequence of the porcine subunit is 82.7% identical to that of the bovine ortholog, but because the density for the N-terminal loop is poorly defined, the porcine model has shortcut some density and has been built in a more extended form.

Subunit c. The bovine and porcine sequences are identical (1, 34). However, the porcine model is incorrect between residues cPro-40 and cGln-45 with cLeu-42 built into the density that should be cLys-43. Whilst the porcine density is inferior to the bovine density in this region, some c-subunits do have unattributed density which accommodates the trimethyl group on residue cLys-43. The revised porcine structure includes the modification (see Fig. S25).

Subunit d. The porcine sequence was assigned but side-chains were truncated to β-carbons. A large piece of density corresponding to residues 147-156 has been short-cut and omitted. The bovine model provides a better explanation of the porcine density. Also, it seems likely that an incorrect version of the sequence has been employed. Uniprot has three different entries for porcine subunit d, and the one used to interpret the porcine density is 137 residues long and lacks residues from 74-97. Entry A0A287A2Y4 is most similar to the bovine sequence. With 161 residues, it is the same length as the bovine subunits with a sequence identity of 89.4%.

Subunit e. The sequences of the porcine and bovine orthologs are 93% identical. The porcine model is similar to the bovine one except that the N-terminal strand (residues 1-7) has been built in the opposite direction into density that probably arises from lipid. Density is present between subunits b and g, as in the bovine data, but it is not connected to the main α-helix density of subunit e. However, when the bovine model was docked into the porcine map, it occupied this density.

Subunit g. Porcine g has been modelled with a similar topology to bovine and yeast orthologs in α-helices gH2 and gH3. However, gH1 was not modelled (Fig. S7 and S8), and density modelled as porcine bH1 is likely to be gH1. Direct comparisons are not possible as the pig density is insufficiently resolved for the sequence register of the protein to be assigned. Parts of porcine gH2 and gH3 have been built into density that should be subunit f. Both the bovine and yeast maps are better resolved in this region.

The peripheral stalk and the OSCP

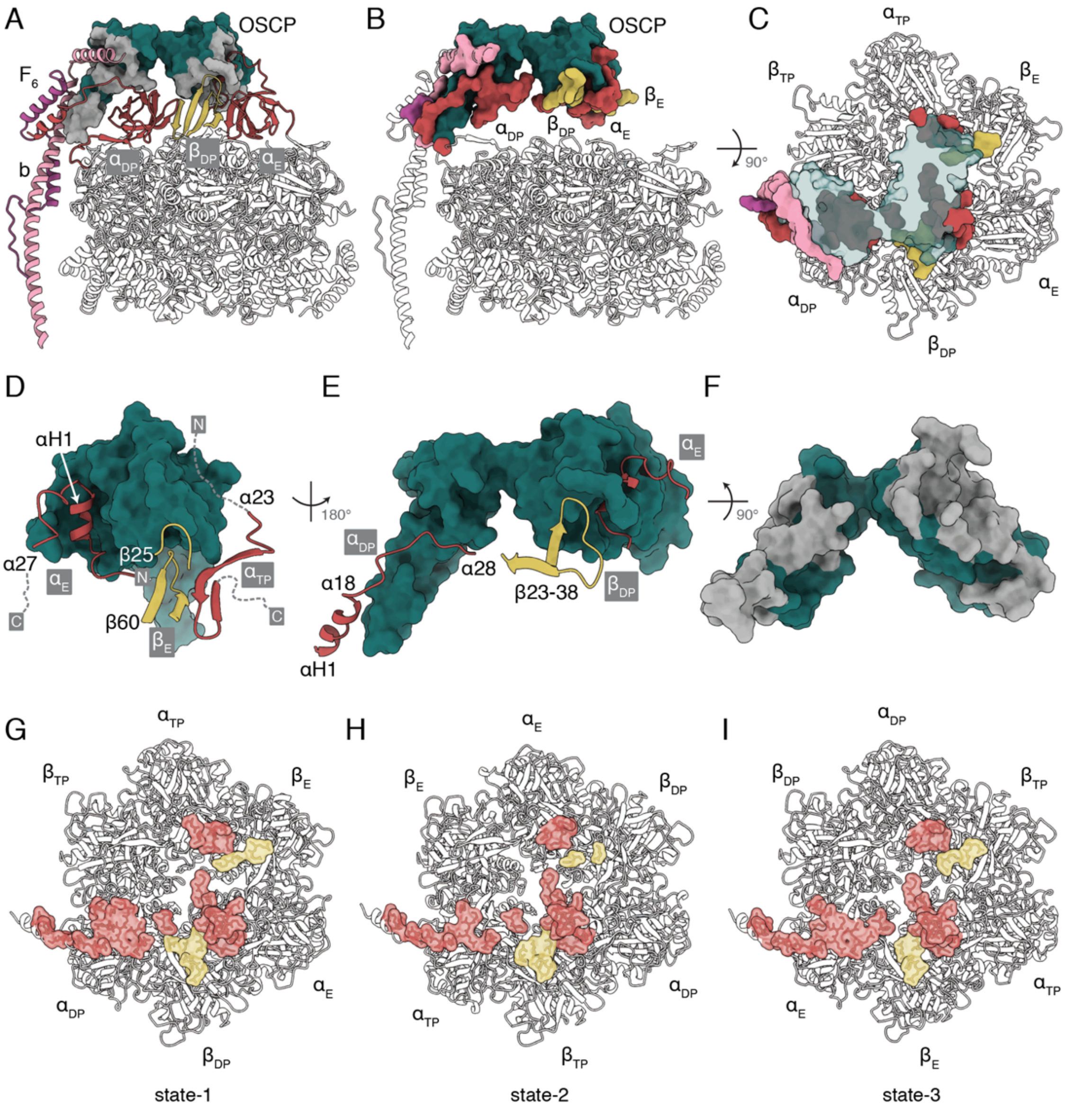

Fig. S19. The cap of the peripheral stalk and changes in its interactions with the crown of the catalytic domain during ATP synthesis by dimeric bovine ATP synthase. The cap is provided by the N-terminal domain of the OSCP. *A*, view of the catalytic domain and the upper region of the PS. The OSCP is shown as a teal solvent excluded molecular surface with residues interacting with adjacent subunits in grey. Interactions between the OSCP and the crown were identified with NCONT, part of the CCP4 suite (35). The coloured structural elements of subunits in the vicinity of the interacting regions of interaction are the C-terminal α-helix of subunit b, the N-terminal α-helix of F_6, and regions of the crown domains of the α_{DP}- and α_E-subunits (red) and the β_{DP}-subunit (yellow). Regions of subunits F_6 are light pink, magenta, respectively. Residues of the crown domains of other α- and β-subunits have been removed for clarity. *B*, the same view showing the molecular surface of the residues in subunits α, β, b and F_6 that interact with the OSCP with adjacent regions in silhouette, and the crown domain removed. *C*, the top of the catalytic domain viewed along the rotary axis, demonstrating the contact patches defined by the residues highlighted in *B* and their relationship to the surface presented by the OSCP (teal transparency). *D* and *E*, details of the interactions between the OSCP and the crown. In *D*, the N-terminal α-helix, αH1, of

the α_E-subunit nestles in a small "pocket" provided residues 1-6 and by α-helices 1 and 5 the OSCP. This seven residue α-helix is followed by a loop (16-28). Two loops, 25-35 and 52-57 of the β_E-subunit, interact slightly with residues 3-6 of the OSCP. Similarly, additional minor contacts are provided by an N-terminal β-strand (residues 28-34) in the α_{TP}-subunit. The cryo-em density preceding this strand, which could not be modelled, appears to progress toward the top of the OSCP, as indicated by a dashed grey line. A similar feature has been observed in fungal ATP synthases. It is probable that the short α-helix is maintained as in the α_{DP}-subunit. However, the poorly defined density suggests that the feature may be mobile and easily dissociated. Given the interactions with the underside surface of the OSCP provided by αH1 in the α_E- and α_{DP}-subunits, and other residues, additional interactions are probably un-necessary for the functioning of the enzyme. *E*, the interaction of the N-terminal region of the α_{DP}-subunit with the OSCP primarily via the loop (residues 19-29) following αH1 and via several residues, disparate in sequence, of the crown domain (see *A-C*). However, the short αH1, appears to make significant contact with residues 11, 15 and 19 of F_6H1 and possibly with residues of the subunit b, thereby making a stable connection to the PS. As in the β_E-subunit, the β_{DP}-subunit shares some minor interactions with OSCP via loops (23-38 and 52-57) in β-strands of the crown domain. *F*, the molecular surface (grey) of OSCP residues that interact with the crown. *G-I*, views along the rotary axis showing changes in the footprint of the OSCP on the crown during rotational states 1, 2 and 3, respectively. The contacts remain relatively constant during rotation. Although the number of residues of chain B of the α-subunit in rotational state 2 appears to reduce (*H*), these contacts return in state 3 (*I*).

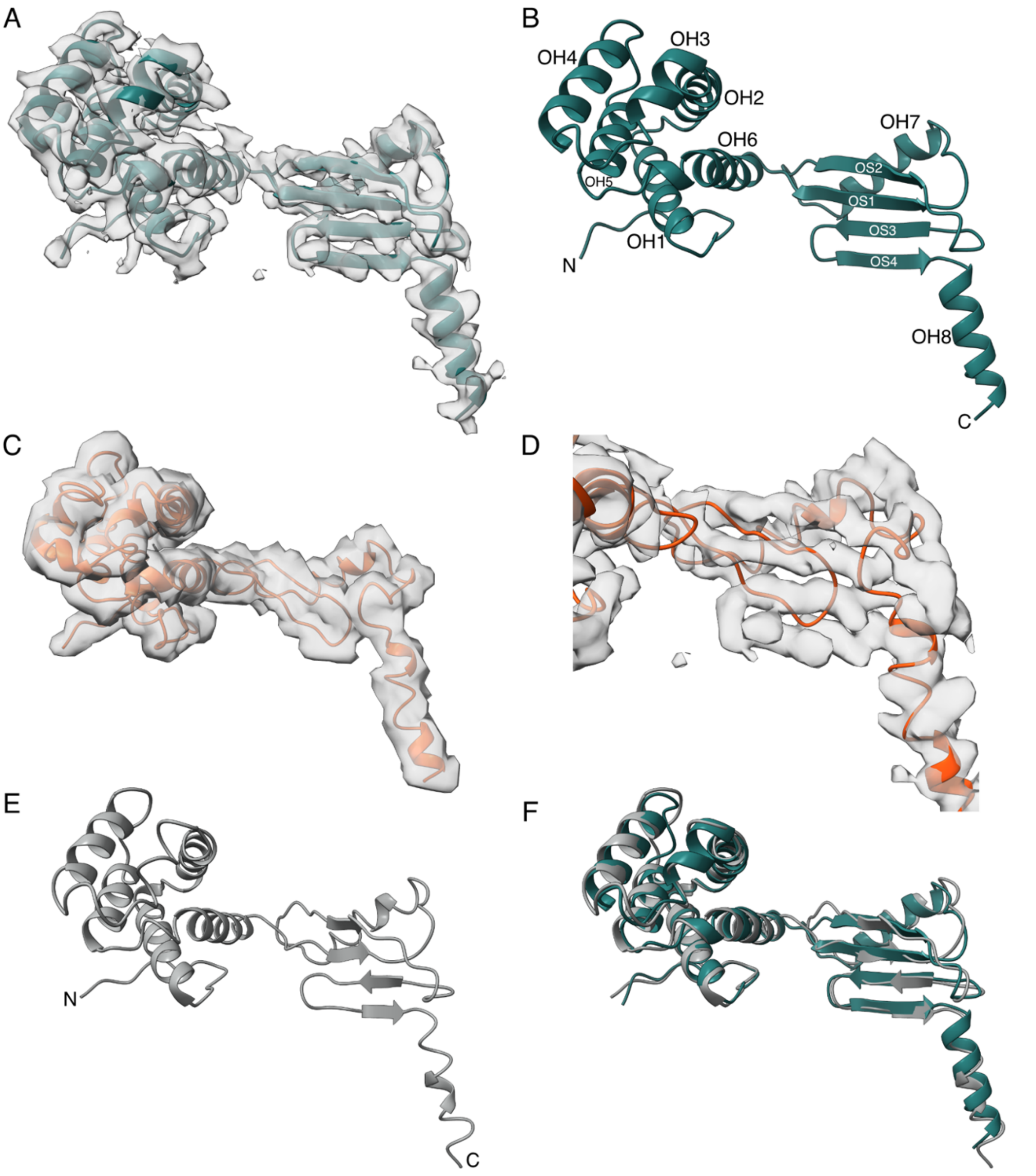

Fig. S20. Resolution of the OSCP subunit of bovine ATP synthase. *A*, electron density map and model of bovine OSCP; *B*, the two domains of the OSCP subunit. The N-terminal domain is a bundle of six α-helices (OH1, residues 14-27; OH2, residues 32-47; OH3, residues 49-56; OH4, residues 62-76; OH5, residues 80-92; and OH6, residues 98-113) which sits on top of the α- and β-subunits, and the C-terminal domain has four β-sheets, (OS1, residues 115-122; OS2, residues 147-155, OS3, residues 162-166; and OS4, residues 169-174), and two α-helices (OH7, residues 128-147; and OH8, residues 175-187), which interact with the PS; *C*, the electron density at 7 Å resolution (EMD-3164) and model of OSCP from monomeric bovine ATP synthase (PDB:5ARA) (23), where the C-terminal domain was interpreted incorrectly; *D*, the model OSCP from the bovine ATP synthase monomer docked into the electron density from the ATP synthase dimer at 3.2 Å resolution. The view has been zoomed in slightly to highlight the improved resolution and definition of the β-sheets. *E*, the model of porcine OSCP (PDB:6J5I) (32) with a similar topology to its bovine

ortholog. Their sequences are 93.7% identical (Fig. S21). *F*, bovine and porcine OSCP subunits superimposed.

```
OSCP 93.68% identity

P13621|ATPO_BOVIN FAKLVRPPVQIYGIEGRYATALYSAASKQNKLEQVEKELLRVGQILKEPKMAASLLNPYV 60
Q2EN81|ATPO_PIG   FAKLVRPPVQIYGIEGRYATALYSAASKQNKLEQVEKELLRVAQILKEPKVAASIMNPYV 60
                  ******************************************.*******:***::****

P13621|ATPO_BOVIN KRSVKVKSLSDMTAKEKFSPLTSNLINLLAENGRLTNTPAVISAFSTMMSVHRGEVPCTV 120
Q2EN81|ATPO_PIG   KRSVKVKSLSDMTAKEKFSPLTSNLINLLAENGRLSSTPGVISAFSTMMSVHRGEVPCSV 120
                  ***********************************:.**.******************:*

P13621|ATPO_BOVIN TTASALDEATLTELKTVLKSFLSKGQVLKLEVKIDPSIMGGMIVRIGEKYVDMSAKTKIQ 180
Q2EN81|ATPO_PIG   TTASPLDEATLTELKTVLKSFLSKGQILKLEVKVDPSIMGGMIVRIGEKYVDMSAKTKIQ 180
                  **** *********************:******:**************************

P13621|ATPO_BOVIN KLSRAMREIL 190
Q2EN81|ATPO_PIG   KLSRAMREIF 190
                  *********:

OSCP 32.8% identity

P13621|ATPO_BOVIN -FAKLVRPPVQIYGIEGRYATALYSAASKQNKLEQVEKELLRVGQ-ILKEPKMAASLLNP 58
P09457|ATPO_YEAST ASKAAAPPPVRLFGVEGTYATALYQAAAKNSSIDAAFQSLQKVESTVKKNPKLGHLLLNP 60
                       . ***:::*:** ******.**:*:..:: . :.* :* . : *:**:.  ****

P13621|ATPO_BOVIN YVKRSVKVKSLSD-MTAKEKFSPLTSNLINLLAENGRLTNTPAVISAFSTMMSVHRGEVP 117
P09457|ATPO_YEAST ALSLKDRNSVIDAIVETHKNLDGYVVNLLKVLSENNRLGCFEKIASDFGVLNDAHNGLLK 120
                   :. . : . :.  : :::::.  . **:::*:**.**     : * *..: ..*.* :

P13621|ATPO_BOVIN CTVTTASALDEATLTELKTVL--KSFLSKGQVLKLEVKIDPSIMGGMIVRIGEKYVDMSA 175
P09457|ATPO_YEAST GTVTSAEPLDPKSFKRIEKALSASKLVGQGKSLKLENVVKPEIKGGLIVELGDKTVDLSI 180
                   ***:*. **  ::..::..*  ..::.:*: ****  :.*.* **:**.:*:* **:*

P13621|ATPO_BOVIN KTKIQKLSRAMREIL 190
P09457|ATPO_YEAST STKIQKLNKVLEDSI 195
```

Fig. S21. Comparison of the sequences of the OSCP subunit in bovine, porcine and yeast ATP synthases. Above, bovine versus porcine; below, bovine versus yeast.

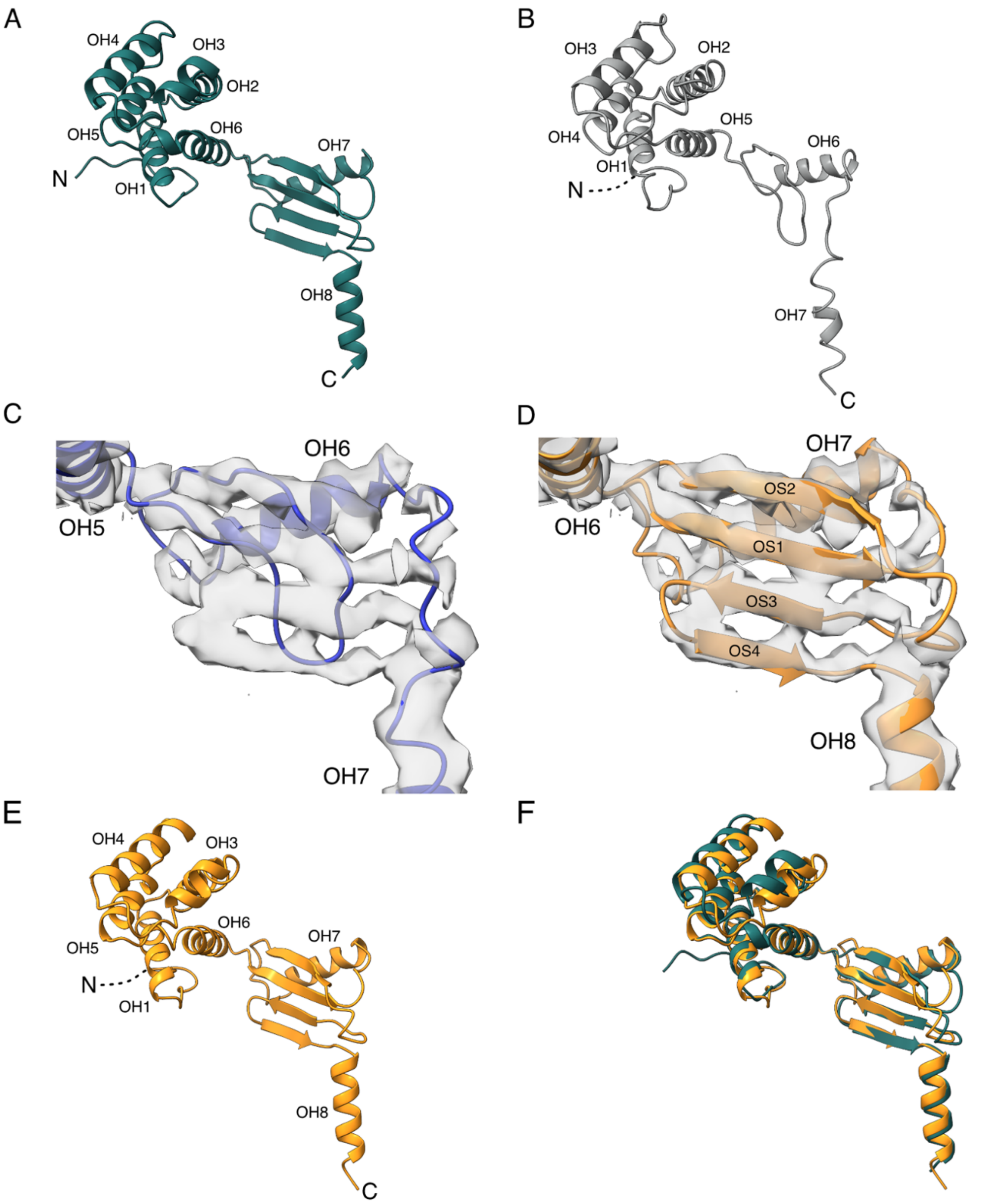

Fig. S22. Comparison of the structures of the OSCP subunits in bovine and yeast ATP synthases. Their sequences are 32.8% identical and the yeast OSCP is five residues longer. The overall topology is similar. However, the published yeast model has been modelled incorrectly. *A*, the bovine OSCP subunit (deep teal) has an N-terminal bundle of six α-helices and a C-terminal domain of four β-sheets and two α-helices (see Fig. S19). *B*, the published model of yeast OSCP (36) (grey), where the equivalent of the short bovine α-helix OH3 is not sufficiently bonded to show as an α-helix and the final α-helix, OH7, is immediately preceded by OH6. *C*, the electron density (EMD-7546) and model of the OSCP from *S. cerevisiae* (PDB:6CP3) (36). The model is based on bovine OSCP (PDB:5ARA) (23). The view has been adjusted to focus on the C-terminal domain.

D, the electron density from *S. cerevisiae* with a revised model of the OSCP subunit from *S. cerevisiae* (orange) with a β-sheet and α-helix motif similar to the bovine ortholog. *E*, the revised model of yeast OSCP, *F*, the revised yeast OSCP subunit (orange) superposed onto the bovine ortholog (deep teal).

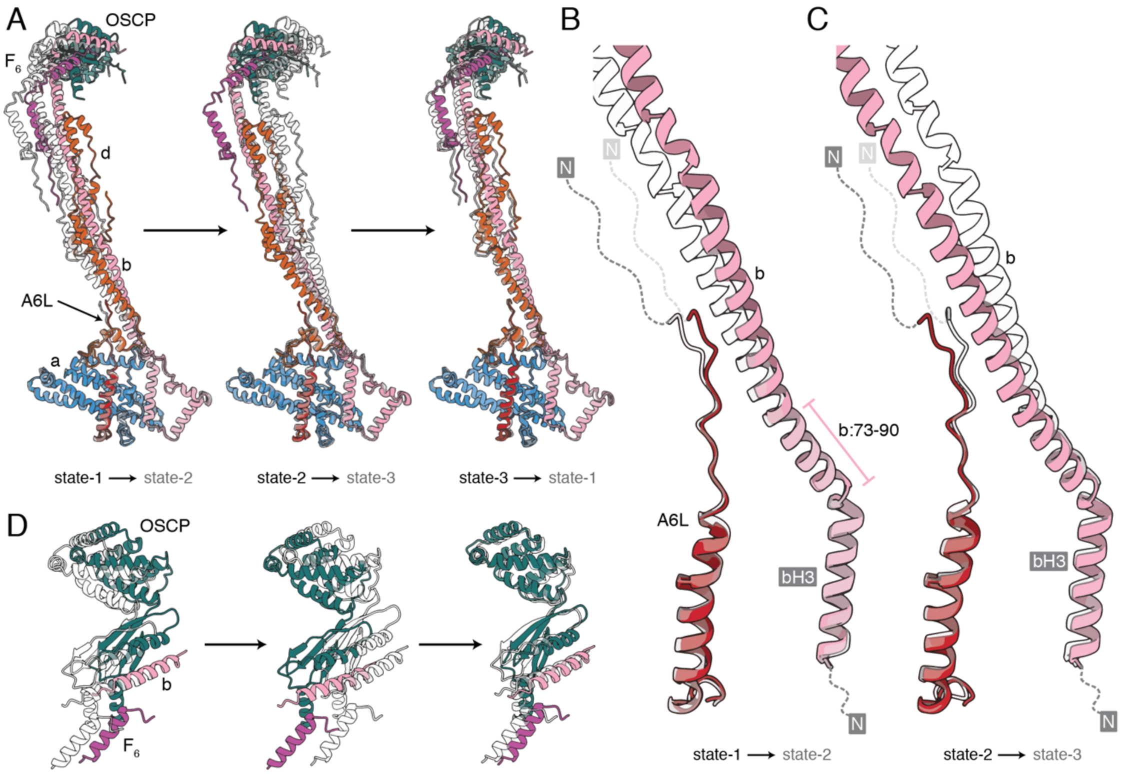

Fig. S23. Movement of the peripheral stalk during the rotary cycle of bovine ATP synthase. *A*, the composite atomic models of rotational states 2 and 3 aligned to the a-subunit of the composite model of rotational state 1. The transitions from state 1 to state 2, from state 2 to state 3 and from state 3 back to state 1 are shown in colour and transparency, respectively in each case. The OSCP, and subunits b, d F_6, A6L and a are teal, light pink, orange, magenta, brick red and cornflower blue, respectively. A significant lateral movement of the entire PS accompanies the rocking motion of the catalytic domain as it and the associated central stalk, progresses through the rotary cycle (see Movie 2). *B*, detail of the hinge region of subunit b, and associated movement of the flexible N-terminal extension of the A6L subunit during the state 1 to state 2 transition. *C*, the same view of the state 2 to state 3 transition. Residues in the region of 73-90 of subunit b remain relatively immobile as bH4, and associated subunits d, F_6 and OSCP, are displaced by 9.9 and 13.1 Å in states 1 and 3, respectively, measured relative to state 2 at the Cα of OSCP G145. *D*, view of the OSCP and associated subunits b and F_6 from above the catalytic domain towards the inner membrane along the axis of rotation of the central stalk. Motions of the catalytic domain are accompanied by movements of the OSCP which is attached to the crown region of the catalytic domain. These movements are transmitted via several contact points including an N-terminal α-helix of the α-subunit (residues 6-18), shown as a red solvent excluded surface in Movie 2, to the PS.

The membrane domain

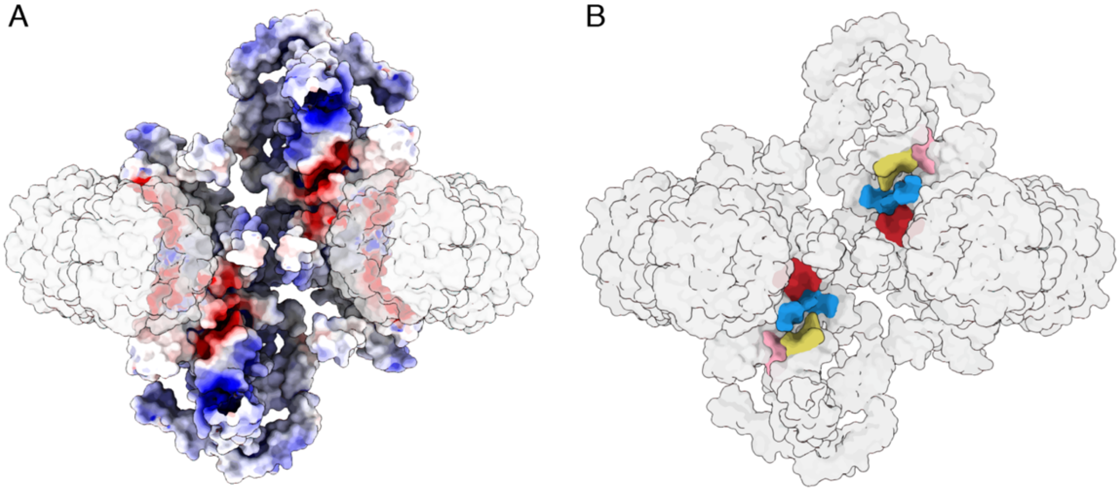

Fig. S24. The electrostatic environment of the IMS surface of the dimeric bovine ATP synthase. *A,* view of the dimeric membrane domain from the IMS. The electrostatic potential of the molecular surface, calculated with the DELPHIpKa web-server (37, 38) at a salt concentration of 150 mM, a pH of 7.4 and otherwise default parameters, is shown with positively and negatively and positively charged surfaces in blue and red, respectively, and the c_8-ring in transparency. The range of charge potentials is -7 (red) to +7 (blue). Residues 41-56 of the e-subunit have been removed for clarity; *B,* the same view with the surfaces of subunits a, A6L, b and f that contribute to the negatively charged areas of the proton inlet channel in cornflower blue, brick red, pink and straw yellow, respectively. The rest of the surface is shown in grey transparency.

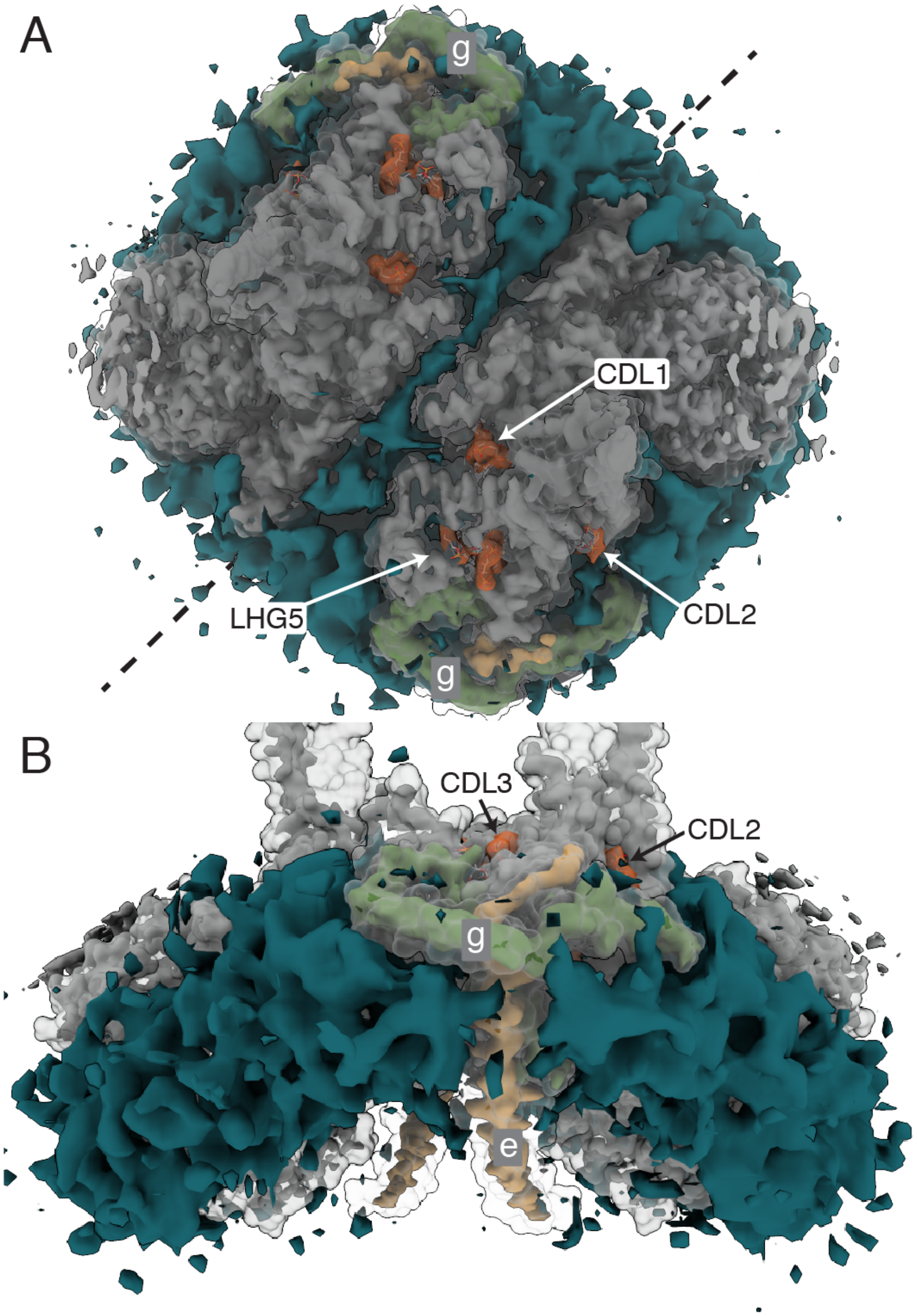

Fig. S25. Lipids in the membrane domain of dimeric bovine ATP synthase. *A* and *B*, the composite cryo-em density of the membrane domain viewed from between the two peripheral stalks and the orthogonal view, respectively. The high-resolution reconstruction of the monomeric membrane domain is grey and subunits e and g are forest green and khaki, respectively. In *A*, the dashed line denotes the interface between the monomers. Cardiolipins CDL1-CD3 and phosphatidylglcerols LH4 and LH5 are resolved (colored orange), as shown also in Figs 1, 2C-D, 4, 5 and S5. The detergent-lipid micelle is teal, and indicates regions between monomers where there are no protein:protein interactions. These regions are occupied by unresolved lipids.

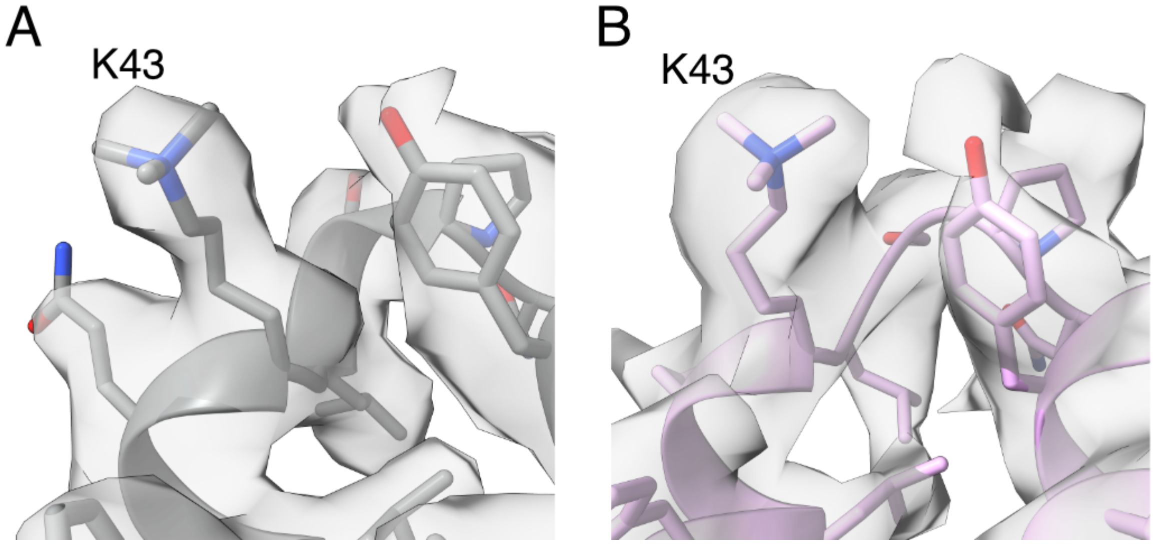

Fig. S26. Density for the trimethyllysine at position 43 of the c-subunit. *A* and *B*, respectively, examples of the density accounted for by the trimethylation of cLys-43 in the bovine and porcine maps. All bovine c-subunits have density consistent with the presence of this modification; *B*, similarly for the porcine map. At least five subunits show the additional density. The model shown is the revised porcine model, the original porcine model having been built incorrectly from residues 40-45.

Table S1. Summary of the structural model of bovine ATP synthase

Subunit	No. of Residues	Residues modelled	Domain	Chain identity
α	510	2-509	F_1	A
α	510	23-401, 411-508	F_1	B
α	510	6-404, 410-508	F_1	C
β	478	9-477	F_1	D
β	478	9-475	F_1	E
β	478	9-475	F_1	F
γ[1]	273	1-272	F_1	G
δ	146	15-146	F_1	H
ε	47	1-47	F_1	I
a (ATP6)	226	3-226	F_o	a
b[2]	214	1-209	F_o	b
c	75	1-75	PS, F_o	c
d[3]	160	5-159	PS	d
e	70	3-58	F_o	e
f	87	5-87	F_o	f
g	102	20-98	F_o	g
j (6.8PL)	60	2-49	F_o	j
k (DAPIT)	57	12-47	F_o	k
A6L (ATP8)	66	1-41	PS, F_o	8
OSCP[4]	190	1-188	PS	S
F_6	76	6-66	PS	h
$IF_{1\ 1\text{-}60}$	60	8-54	F_1	J

[1] Residues 62-66 form an extended loop and residues 97-100 extend an α-helix by two residues followed by two residues of loop; previously both regions were unresolved; [2] residues 1-121 and 208-209 were resolved in addition to the previously known structure (PDB:2CLY); [3] residues 124-159 were resolved in addition to the previously determined structure (PDB:2CLY); [4] published models and predicted secondary structures of the C-terminal domain were inconsistent (Fig. S19). The present structure shows that the C-terminal domain consists of four β-strands with an α-helix between strands 1 and 2 and a second α-helix in the C-terminal region. In the PS, these α-helices link the OSCP to the b-subunit. The structure of the OSCP in the model of yeast ATP synthase was also incorrect in the C-terminal domain and the data have been re-interpreted (Fig. S21).

Table S2. Deposited data-sets relating to the structure of the dimeric bovine ATP synthase

	PBD	EMDB	Resolution (Å)	B-factor ($Å^2$)	Num. particles	Detail	Comment
**State 1**	6YY0	EMD-11001	3.23	-51	101,165	ATP synthase monomer in rotational state 1 (Scheme 1)	Best F_1-OSCP density for state, facilitated modelling of catalytic domain
**State 2**	6Z1R	EMD-11039	3.29	-42	90,850	ATP synthase monomer in rotational state 2 (Scheme 1)	〃
**State 3**	6Z1U	EMD-11040	3.47	-52	61,458	ATP synthase monomer in rotational state 3 (Scheme 1)	〃
**membrane**	6ZBB	EMD-11149	3.61	-105	253,473	Local refinement of all monomer particles focussed on the membrane domain (Scheme 2)	Best membrane domain density, facilitated modelling of all membrane intrinsic subunits
**State 1-rotor**	6ZG7	EMD-11195	3.49	-98	101,165	Local refinement of state-1 monomer particles focussed on subunits γ, δ, ε and c-ring (Scheme 2)	Homogenous c-ring, improved γ, δ, ε
**State 2-rotor**	6ZG8	EMD-11196	3.49	-90	90,850	Local refinement of state-2 monomer particles focussed on subunits γ, δ, ε and c-ring (Scheme 2)	〃
**State 3-rotor**	6ZIK	EMD-11227	3.66	-102	61,458	Local refinement of state-3 monomer particles focussed on subunits γ, δ, ε and c-ring (Scheme 2)	〃
**State 1-PS**	6ZIQ	EMD-11228	4.22	-135	101,165	Local refinement of state-1 monomer particles focussed on subunits b, d, OSCP and A6L, a, e, f, g, j and k (Scheme 2)	Improved continuity of b-subunit density, best PS map, facilitated modelling of subunits b, d and F_6

**State 2-PS**	6ZIT	EMD-11229	4.33	-156	90,850	Local refinement of state-2 monomer particles focussed on subunits b, d, OSCP and A6L, a, e, f, g, j and k (Scheme 2)	Poorer quality, OK for rigid body fit
**State 3-PS**	6ZIU	EMD-11230	6.02	-191	61,458	Local refinement of state-3 monomer particles focussed on subunits b, d, OSCP and A6L, a, e, f, g, j and k (Scheme 2)	Poorer quality, OK for rigid body fit
**State 1-composite**	6ZPO	EMD-11342	3.23-4.22	n/a	n/a	phenix.combine_focused_maps Ref: state-1	possible errors in subunit j position (due to mask proximity)
**State 2-composite**	6ZQM	EMD-11368	3.29-4.33	n/a	n/a	phenix.combine_focused_maps Ref: state-2	〃
**State 3-composite**	6ZQN	EMD-11369	3.47-6.02	n/a	n/a	phenix.combine_focused_maps Ref: state-3	〃
**Porcine F_o**	6ZMR	EMD-0668	3.94			Re-interpreted and refined porcine F_o model	
**Porcine F_o tetramer**	6ZNA	EMD-0667	6.2			Re-interpreted F_o domains docked into porcine tetramer map EMD-0667	Sidechains trimmed to Cβ

Table S3. Model refinement statistics

Model	PDB ID	EMDB ID	RMS bonds		Ramachandran		Rotamer	Molprobity	Clashscore	EMRinger
			length (Å)	angles (°)	outliers (%)	favoured (%)	outliers (%)	score		score
State 1 F_1	6YY0	EMD-11001	0.006	0.626	0.0	91.55	0.0	1.94	8.07	2.97
State 2 F_1	6Z1R	EMD-11039	0.007	0.658	0.0	93.35	0.0	1.91	9.02	2.88
State 3 F_1	6Z1U	EMD-11040	0.007	0.687	0.0	91.10	0.0	2.09	11.32	2.33
F_o all states	6ZBB	EMD-11149	0.006	0.710	0.0	93.94	0.0	2.11	15.86	1.49
State 1 rotor	6ZG7	EMD-11195	0.006	0.641	0.0	95.22	0.0	1.85	9.98	2.53
State 2 rotor	6ZG8	EMD-11196	0.006	0.668	0.0	96.10	0.0	1.86	11.93	2.19
State 3 rotor	6ZIK	EMD-11227	0.005	0.706	0.0	97.91	0.0	1.54	9.91	2.19
State 1 PS	6ZIQ	EMD-11228	0.006	0.794	0.07	89.05	0.0	2.40	21.46	0.89
State 2 PS	6ZIT	EMD-11229	0.005	0.740	0.0	89.40	0.0	2.40	22.0	0.69
State 3 PS	6ZIU	EMD-11230	0.004	0.753	0.0	89.54	0.0	2.49	27.65	0.43
Porcine F_o E state	6ZMR	EMD-0668	0.006	0.778	0.0	90.61	0.0	2.24	16.09	1.54

Movie legends

Movie S1 (separate file) The structure of dimeric bovine ATP synthase. The monomeric structure is built up from individual subunits depicted as cryo-em densities. The PS components are added to the F_1-c_8 subcomplex, and then the view is adjusted to show the membrane domain, and addition of the supernumerary subunits. The view is readjusted and the dimer is formed by the addition of the second monomer. The detergent/lipid micelle observed in the data is also shown for reference. The α-, β-, γ-, δ- and ε-subunits of the F_1-catalytic domain are red, yellow, blue, indigo and green, respectively, with the central stalk (subunits γ, δ and ε) attached to the c_8-ring (dark grey) in the membrane domain in contact with subunit a or ATP6 (cornflower blue). The PS subunits, OSCP, b, d and F_6 are teal, light pink, orange and magenta, respectively, and the A6L subunit is brick red. In the region of the monomer-monomer interface, subunits e, f, g, j and k are khaki, straw yellow, forest green, sea-foam green and dark pink, respectively. Cardiolipin (CDL) and phosphatidyl-glycerol (LHG) phosphate headgroups are scarlet and acyl chains grey.

Movie S2 (separate file) Lateral movements of the PS associated with the rotary cycle of bovine ATP synthase. This movie demonstrates the extensive lateral movements of the PS and the rocking motion of the catalytic domain as the asymmetric central stalk rotates within the F_1-domain. The movie illustrates mechanical motions in the peripheral stalk, but much of the nuance accompanying rotation, including rotary sub-steps, is lost because of the use of the inhibitor protein to lock in enzyme in a defined catalytic state. The motions of the PS may proceed stepwise with the corresponding sub-steps or may move continuously. However, the relatively poor map quality in this region could suggest residual heterogeneity of a continuous nature that is often unresolved by the methods of image classification used here. The catalytic domain and rotor are shown as a grey solvent excluded surface. Residues 1-25 of the α-subunits are dull red. Subunits A6L, a, b, d F_6 and the OSCP in cartoon representation are brick red, cornflower blue, light pink, orange, magenta and teal, respectively. At the end of the sequence, a top view along the rotary axis demonstrates the coupling of the F_1-domain to the PS, via the OSCP, which follows the rocking motion of the catalytic domain. The N-termini of the α-subunits are dull red and can be seen bound to both the OSCP and regions of the PS subunits b and F_6.

Movie S3 (separate file) The universal joint provided by the OSCP. The movie depicts the conformational changes in the OSCP during the transition between catalytic states. The sequence is viewed top-down along the rotary axis. In addition to the global displacement of the OSCP during the catalytic cycle, the subunit also demonstrates a hinge-like conformational change about the loop (residues 112-116) connecting the N- and C-terminal domains. In the state 1:state 2 and state 2:state 3 transitions, the C-terminal domain moves as an approximately rigid body, associated with alterations to β-strand 2 (residues 148-154), the loop (residues 155-160) connecting β-strand 2 to β-strand 3 (residues 161-166) and the loop connecting α-helix 7 (residues 91-129) to β-strand 2. In the state 3:state 1 transition, the C-terminal domain is not laterally displaced, but it expands away from the PS as the domain is put under tension.

SI References

1. M.J. Runswick *et al.*, The affinity purification and characterization of ATP synthase complexes from mitochondria. *Open Biol* **3**, 120160 (2013).
2. U.K. Laemmli, Cleavage of structural proteins during the assembly of the head of bacteriophage T4. *Nature* **227**, 680-685 (1970).
3. H. Schägger, G. von Jagow, Blue native electrophoresis for isolation of membrane protein complexes in enzymatically active form. *Anal. Biochem.* **199**, 223-231 (1991).
4. J.R. Meyerson *et al.*, Self-assembled monolayers improve protein distribution on holey carbon cryo-EM supports. *Sci Rep* **4**, 7084 (2014).

5. S. Chen *et al.*, High-resolution noise substitution to measure overfitting and validate resolution in 3D structure determination by single particle electron cryomicroscopy. *Ultramicroscopy* **135**, 24-35 (2013).
6. S.H. Scheres, RELION: implementation of a Bayesian approach to cryo-EM structure determination. *J. Struct. Biol.* **180**, 519-530 (2012).
7. S.H. Scheres, A. Bayesian view on cryo-EM structure determination. *J. Mol. Biol.* **415**, 406-418 (2012).
8. S.Q. Zheng *et al.*, MotionCor2: anisotropic correction of beam-induced motion for improved cryo-electron microscopy. *Nat Methods* **14**, 331-332 (2017).
9. A. Rohou, N. Grigorieff, CTFFIND4: Fast and accurate defocus estimation from electron micrographs. *J. Struct. Biol.* **192**, 216-221 (2015).
10. T. Wagner *et al.*, SPHIRE-crYOLO is a fast and accurate fully automated particle picker for cryo-EM. *Commun Biol* **2**, 218 (2019).
11. R. Fernandez-Leiro, S.H.W. Scheres, A pipeline approach to single-particle processing in RELION. *Acta Crystallogr D Struct Biol* **73**, 496-502 (2017).
12. J. Zivanov *et al.*, New tools for automated high-resolution cryo-EM structure determination in RELION-3. *Elife* **7**, (2018).
13. J.R. Gledhill, M.G. Montgomery, A.G.W. Leslie, J.E. Walker, How the regulatory protein, IF_1, inhibits F_1-ATPase from bovine mitochondria. *Proc. Natl. Acad. Sci. U. S. A.* **104**, 15671-15676 (2007).
14. J. Zivanov, T. Nakane, S.H.W. Scheres, Estimation of high-order aberrations and anisotropic magnification from cryo-EM data sets in RELION-3.1. *IUCrJ* **7**, 253-267 (2020).
15. P. Emsley, B. Lohkamp, W.G. Scott, K. Cowtan, Features and development of Coot. *Acta Crystallogr D Biol Crystallogr* **66**, 486-501 (2010).
16. D. Liebschner *et al.*, Macromolecular structure determination using X-rays, neutrons and electrons: recent developments in Phenix. *Acta Crystallogr D Struct Biol* **75**, 861-877 (2019).
17. P.V. Afonine *et al.*, Real-space refinement in PHENIX for cryo-EM and crystallography. *Acta Crystallogr D Struct Biol* **74**, 531-544 (2018).
18. P.V. Afonine *et al.*, New tools for the analysis and validation of cryo-EM maps and atomic models. *Acta Crystallogr D Struct Biol* **74**, 814-840 (2018).
19. T.I. Croll, ISOLDE: a physically realistic environment for model building into low-resolution electron-density maps. *Acta Crystallogr D Struct Biol* **74**, 519-530 (2018).
20. V.K. Dickson, J.A. Silvester, I.M. Fearnley, A.G.W. Leslie, J.E. Walker, On the structure of the stator of the mitochondrial ATP synthase. *EMBO J.* **25**, 2911-2918 (2006).
21. I.N. Watt, M.G. Montgomery, M.J. Runswick, A.G.W. Leslie, J.E. Walker, Bioenergetic cost of making an adenosine triphosphate molecule in animal mitochondria. *Proc. Natl. Acad. Sci. U. S. A.* **107**, 16823-16827 (2010).
22. D.M. Rees, A.G.W. Leslie, J.E. Walker, The structure of the membrane extrinsic region of bovine ATP synthase. *Proc. Natl. Acad. Sci. U. S. A.* **106**, 21597-21601 (2009).
23. A. Zhou *et al.*, Structure and conformational states of the bovine mitochondrial ATP synthase by cryo-EM. *Elife* **4**, e10180 (2015).
24. V.B. Chen *et al.*, MolProbity: all-atom structure validation for macromolecular crystallography. *Acta Crystallogr D Biol Crystallogr* **66**, 12-21 (2010).
25. I.W. Davis *et al.*, MolProbity: all-atom contacts and structure validation for proteins and nucleic acids. *Nucleic Acids Res.* **35**, W375-83 (2007).
26. B.A. Barad *et al.*, EMRinger: side chain-directed model and map validation for 3D cryo-electron microscopy. *Nat Methods* **12**, 943-946 (2015).
27. F. Madeira *et al.*, The EMBL-EBI search and sequence analysis tools APIs in 2019. *Nucleic Acids Res.* **47**, W636-W641 (2019).
28. D.T. Jones, Protein secondary structure prediction based on position-specific scoring matrices 1 1Edited by G. Von Heijne. *J. Mol. Biol.* **292**, 195-202 (1999).
29. D.W.A. Buchan, D.T. Jones, The PSIPRED Protein Analysis Workbench: 20 years on. *Nucleic Acids Res.* **47**, W402-W407 (2019).

30. T.D. Goddard *et al.*, UCSF ChimeraX: Meeting modern challenges in visualization and analysis. *Protein Sci* **27**, 14-25 (2018).
31. J. Lee *et al.*, Organization of Subunits in the Membrane Domain of the Bovine F-ATPase Revealed by Covalent Cross-linking. *J. Biol. Chem.* **290**, 13308-13320 (2015).
32. J. Gu *et al.*, Cryo-EM structure of the mammalian ATP synthase tetramer bound with inhibitory protein IF1. *Science* **364**, 1068-1075 (2019).
33. H. Guo, SA Bueler, JL Rubinstein, Atomic model for the dimeric F_o region of mitochondrial ATP synthase. *Science* **358**, 936-940 (2017).
34. T.B. Walpole *et al.*, Conservation of complete trimethylation of lysine-43 in the rotor ring of c-subunits of metazoan adenosine triphosphate (ATP) synthases. *Mol Cell Proteomics* **14**, 828-840 (2015).
35. M.D. Winn *et al.*, Overview of the CCP4 suite and current developments. *Acta Crystallogr D Biol Crystallogr* **67**, 235-242 (2011).
36. A.P. Srivastava *et al.*, High-resolution cryo-EM analysis of the yeast ATP synthase in a lipid membrane. *Science* **360**, (2018).
37. S. Sarkar *et al.*, DelPhi Web Server: A Comprehensive Online Suite for Electrostatic Calculations of Biological Macromolecules and Their Complexes. *Communications in Computational Physics* **13**, 269-284 (2013).
38. N. Smith *et al.*, DelPhi web server v2: incorporating atomic-style geometrical figures into the computational protocol. *Bioinformatics* **28**, 1655-1657 (2012).
